# Geophagy in Gibraltar Barbary macaques is a primate tradition anthropogenically induced

**DOI:** 10.1038/s41598-026-44607-0

**Published:** 2026-03-19

**Authors:** J. Frater, M. Nicourt, F. Landi, B. Maxwell, J. Thiodet, E. Mestrallet, S. J. Warr, M. Pizarro, J. E. Fa, S. Lemoine

**Affiliations:** 1 Department of Archaeology, University of Cambridge, Cambridge, UK; 2 Wildlife Conservation Research Unit, University of Oxford, Department of Biology, Life and Mind Building, Oxford, UK; 3 Faculté des Sciences et de l’Ingénierie, University of Paris-Sorbonne, Paris, France; 4 Institut Català de Paleoecologia Humana i Evolució Social, Tarragona, Spain; 5 Research Unit, University of Gibraltar, Gibraltar, UK; 6 UFR Lettres, Langues, Sciences Humaines et des Sociétés, University Sorbonne Paris-Nord, Villetaneuse, France; 7 AGROPARISTECH, Paris Saclay University, Gif-sur-Yvette, France; 8 Department of the Environment, Sustainability, Climate Change and Heritage, HM Government of Gibraltar, Gibraltar, UK; 9 Manchester Metropolitan University, Manchester, UK

**Keywords:** Barbary macaques, Geophagy, Protection and supplementation hypotheses, Animal culture, Anthropogenic context, Human-primate interface, Ecology, Ecology, Evolution, Zoology

## Abstract

We report, for the first time, geophagy – the deliberate consumption of earth – in the Barbary macaque (*Macaca sylvanus*) population living at the human-primate interface in Gibraltar. We evaluate potential adaptive functions of this behavior in an anthropogenic context, drawing on predictions from the protection and supplementation hypotheses. Geophagy occurred at exceptionally high rates compared to other macaque species and locations, and it was more common in summer when tourist numbers peak. It was also more likely when macaques consumed greater amounts of tourist-derived food, supporting a protective function. Local ecological factors contributed as well, with the distribution of red soil (terra rossa) influencing geophagy frequency. Although the behavior was not linked to female reproductive status, supplementation cannot be fully dismissed given the very limited insectivory in this population. We propose that tourist-derived foods may disrupt gut microbiome composition, producing discomfort that individuals mitigate through soil ingestion. Geophagy is likely socially learned, as groups showed consistent preferences for specific soil types, and its practice in presence of conspecifics offered opportunities for social learning. Reports from other sites indicate that geophagy is not unique to Gibraltar, but in this population it appears to be anthropogenically induced and socially transmitted, forming a locally maintained tradition.

**Supplementary Information:**

The online version contains supplementary material available at 10.1038/s41598-026-44607-0.

## Introduction

Geophagy—the intentional and non-accidental consumption of earth materials such as soil, chalk and clay—is common across the animal kingdom, documented in birds, large-bodied mammals, but also non-human primates and humans^1–5^. Its functional significance remains debated, with several non-mutually exclusive hypotheses^3,5^. The detoxification/protection hypothesis proposes that geophagy is a form of medication, used to reduce the short-term malaise and long-term effects of harmful chemicals and parasites and pathogens^5^. Under this hypothesis, geophagy may be protective by reducing the permeability of the gut wall to toxins and pathogens and by binding directly to those^5,6^, hereby adsorbing toxins, but also modulating gut pH, alleviating diarrhea, and generally protecting against endoparasites^2,7^.

The nutrient deficiency/supplementation hypothesis proposes that geophagy may function as a source of essential nutrients otherwise absent or limited in the diet (e.g. iron, sodium, calcium, magnesium…)^2,5^. This has been particularly highlighted for iron deficiencies in humans where anemia is frequently associated with geophagy^5,8^. It has also been proposed that sodium deficiencies may drive geophagy in non-human animals^2^.

The non-adaptive hypothesis of geophagy suggests that it may confer no substantial benefit, and in some cases could be detrimental. Earth components are consumed when there is no food to eat, arising from hedonic cravings in humans^9^ or due to neurological problems arising from micronutrients deficiencies, making non-food items become appealing^5,10^. In non-human animals, and in particular in primates, non-adaptive explanations of geophagy rather consider it as a feeding custom not conferring adaptive benefits, and constituting a cultural tradition as in gorillas^11^. In non-human primates, geophagy is a common phenomenon, occurring in more than half of primate genera spanning a diversity of social systems, diet, and taxonomic units (reviewed in^2,3^. Being particularly practiced in herbivorous and folivorous species across mammals^12,13^, geophagy may have facilitated plants consumption by mitigating the negative effects of secondary compounds, which can be bound and neutralized by earth minerals, especially clay^12,13^. In non-human animals, and across the primate lineage in particular, this behavior may have facilitated the adaptability to novel diets and environments, representing an early form of self-medication^5,14,15^. Geophagy in non-human primates is adaptive in several ways, supporting both the protection and supplementation hypotheses, although with more consistent support for the protection, keeping in mind the caveats in data availability and consistency across studies^3^.

Despite growing interest in primate forms of self-medication^16^, and in geophagy^2,3^, these behaviors are rarely placed into the context of rapidly changing environmental conditions. Human–primate interfaces exemplify this context, being environments where humans and non-human primates interact directly or indirectly, hereby influencing the behavior, ecology, health, or conservation of primates^17^. Due to some primates’ behavioral flexibility, such contexts can promote novel, adaptive behaviors often linked to foraging and new dietary niches^18^. Examples of novel foraging behavior include bottle cap opening in Indian bonnet macaques (*Macaca radiata*)^19^ and robbing-bartering in Balinese long-tailed macaques (*M. fascicularis)*^20^. Gibraltar provides a striking example of a human-primate interface, where a semi-wild population of Barbary macaque (*M. sylvanus*) attracts thousands of visitors annually^21^(e.g. 859,092 visitors to the Upper Rock Nature Reserve in 2024, https://www.gibraltar.gov.gi/). Tourist sites vary in visitor density and types of human-macaque interactions, creating a spatio-temporal anthropogenic gradient. The macaque population, ca. 230 individuals in eight permanent groups, is provisioned daily with food and water by local authorities^22^. Although tourists are forbidden to feed or touch the primates, such interactions are common. Despite decades of study in this population – though never continuously - little attention has focused on novel behaviors emerging from sustained human contact, whether these behaviors are adaptive, or their health and conservation implications.

We present the first detailed account of geophagy in the Gibraltar Barbary macaque population. Although some Gibraltarians are aware of this behavior, it has not been formally recorded in this population or species^3^. Following guidelines proposed by^3^, we first describe population-wide patterns of geophagy, including its distribution across groups, sexes, seasons, anthropogenic and social contexts. We then quantitatively test predictions from the protection and supplementation hypotheses to assess the functional significance of geophagy in this population and anthropogenic context. Here, we consider that geophagy can be defined as a cultural habit if it is socially learned and transmitted across individuals and generations^23^ and if its prevalence and patterns differ between groups and populations^24^. Thus, geophagy as a socially learned behavior may not necessarily be mutually exclusive from adaptive explanations such as protection and/or supplementation,

Gibraltar macaques mainly eat fruits, vegetables and seeds that are provided at designated feeding platforms by the management (provisioned food), but also a substantial amount of items obtained from visitors (tourist-derived food), and only smaller amounts of foods naturally foraged (natural food)^25^. We hypothesize that geophagy is linked to ingesting tourist-derived foods, which may apply to both protection and supplementation hypotheses. The protection hypothesis proposes that geophagy provides protection against toxins found in the diet and environment. We expand this hypothesis to the anthropogenic context and propose that tourist-derived foods may disrupt gut function and trigger soil consumption as a protective response. Human-based diets can impair non-human primate gastrointestinal function^26^, including altering the gut microbiome^27^. In Gibraltar, tourist-derived foods are typically rich in calories, sugar, salt and dairy, but low in fiber. These conditions could promote geophagy as a buffering mechanism. Under the protection hypothesis, geophagy should be less frequent when macaques eat more non-transformed food - provisioned by the management such as seeds, vegetables and fruits, and naturally foraged such as grass, piths and olive seeds - and more frequent when their diet includes more tourist-derived items, such as crisps, chocolate bars, ice-cream etc… The supplementation hypothesis proposes that geophagy compensates for mineral or nutrient deficiencies in the regular diet. In the Gibraltar anthropogenic context, this hypothesis then also predicts higher geophagy when individuals consume less non-transformed food that should contain all essential nutrients, and thus more geophagy when more tourist-derived food are ingested if those lack of essential nutrients. In contrast to the protection hypothesis, the supplementation hypothesis also predicts increased geophagy during nutritionally demanding periods such as pregnancy and lactation. If geophagy is not related to tourist-derived food, we expect no association between its rate and amounts of food types (tourist-derived or non-transformed).

We also test whether geophagy in Gibraltar macaques has become part of the species’ cultural repertoire and, possibly, is specific to this population, which would be indicative of a behavior socially learned and transmitted. Socially learned behavioral traits that are population specific are considered as cultural traits in animal studies^23^, and group-specific feeding preferences have been shown to reflect social learning in humans^28^ and non-human primates^29–31^. Alongside contextual observations of geophagy and its social practice, and in absence of sufficient data to conduct diffusion analyses, we conducted experiments offering individuals four types of locally available soils previously seen being consumed to assess group-specific preferences. If geophagy in Gibraltar is socially learned, we predicted soil preferences would match each group’s observed feeding patterns. To assess how widespread this behavior is, we surveyed researchers and specialists working with Barbary macaques across free-ranging, semi–free-ranging, and captive populations. This allowed to determine whether geophagy is widespread within the species or distinctive to Gibraltar. Finally, we examined ecological drivers that might shape this feeding tradition, testing whether geophagy reflects necessity – such as a compensatory response to ingesting tourist-derived food –– or opportunity, in which it emerges in areas with high density of soil outcrops. Framing geophagy in terms of necessity versus opportunity^32^ clarifies how local ecology influences culturally transmitted behaviors.

## Results

### Distribution of events across observation periods and soil types

**Fig. 1:**
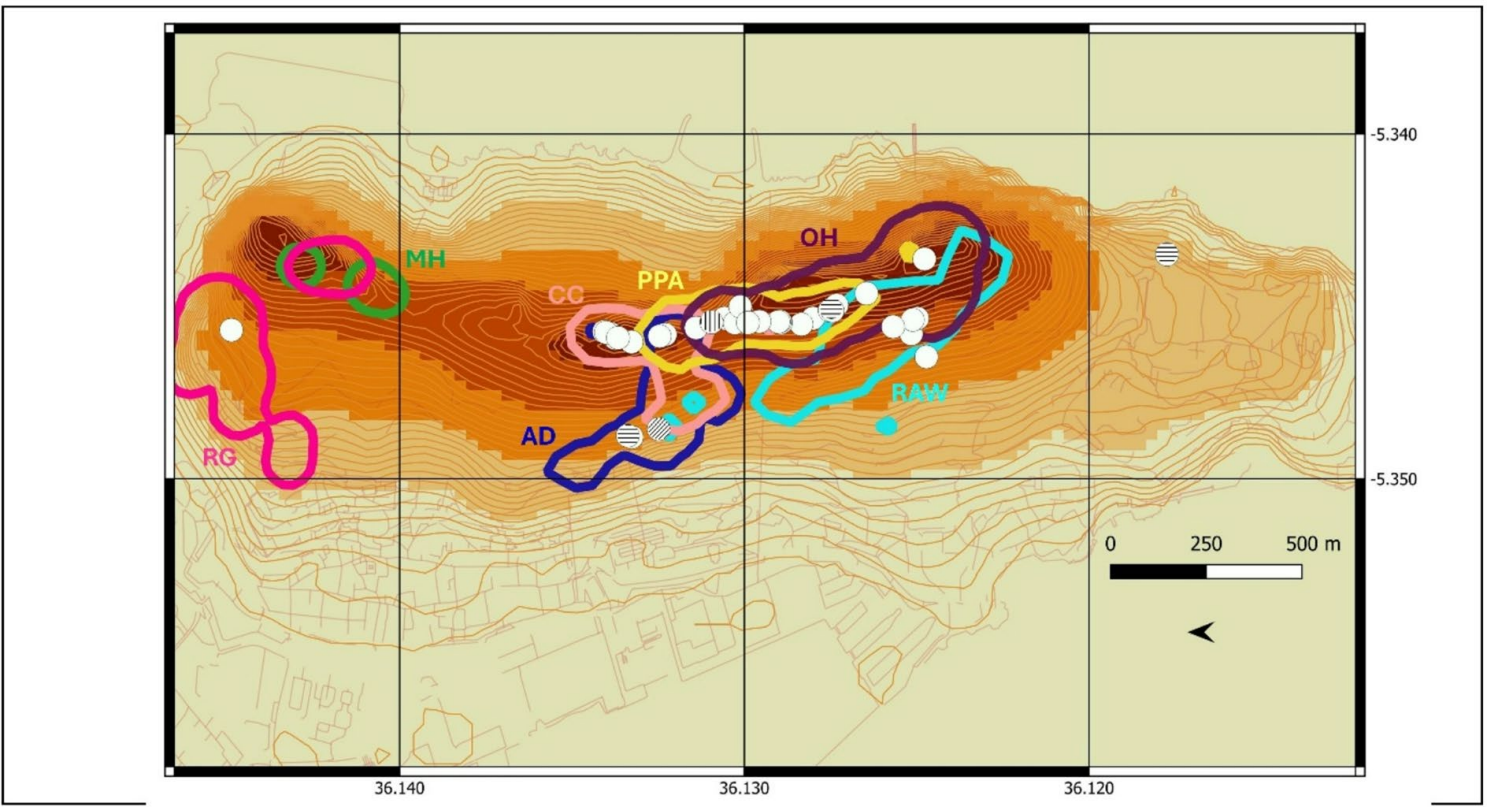
Elevation gradient and macaque home-ranges in Gibraltar. Elevation gradient is depicted in shades of orange (darker colors are higher), home ranges of the main study groups are delimited by 95% kernel utilization distributions: Apes Den (AD) in dark blue; Royal Anglian Way (RAW) in light blue; Cable Car (CC) in light pink; Prince Philip Arch (PPA) in yellow; O’Hara (OH) in purple; Middle Hill (MH) in green; Rock Gun (RG) in dark pink. White dots, horizontally shaded dots, vertically shaded dots, and oblique shade dots correspond to the recorded events of geophagy on red soil, black soil, yellow soil and tar, respectively. This map has been created using QGIS Desktop 3.38.3 Grenoble https://www.qgis.org.

Between 19 August 2022 and 12 April 2024, across five field seasons and a total of 98 observation days, we recorded 46 geophagic events in the macaque population performed by 44 distinct individuals (20 during individual focal follows by the focal individual, 18 during *ad-libitum* sampling including 7 during focal follows but by other individuals, and eight during group scans), observed in 31 distinct days. Figure 1 shows the spatial distribution of all events. Of the 46 cases, 38 occurred on red soil (hereafter called terra rossa) outcrops (82.6%), three on yellow soil (6.5%), three on black soil (6.5%) and two on tar (4.3%).

### Ingestion patterns

When ingesting red, yellow and black soil, individuals typically stopped at ground-level outcrops while traveling. Sitting on the ground or standing quadrupedally against the rock face, they manually collected pieces of soil and brought them to their mouth. For tar ingestion, individuals repeatedly used the same asphalt road hole, manually gathering small fragments (ca. 2 mm) or breaking off large pieces before ingesting them. We could not determine whether material was swallowed directly or temporally stored in cheek pouches. Careful inspection of ingestion sites revealed no seeds, insects or eggs, and video analyzes (30 of 46 cases) confirmed these events were not misidentified foraging. Geophagy actions, characterized by goal-directed motions and precision grip to pick small elements, contrasted consistently with ground-feeding postures and actions such as quadrupedal motion foraging, ground digging and hand-sweeping motions.

Although exact quantities could not be measured, video footage showed intake ranging from millimeter-sized fragments to larger pieces several centimeters across (Movies S1 and S2). Of the 46 cases, 17 had full video sequences allowing quantification. On average, geophagy lasted 20.74 ± 11.7 s (range: 5–45.1 s), with 4.53 ± 2.4 intakes per event (range: 1–11). Intake rates averaged 15 items per minute (± 7.3; range: 6.4–36).

### Inter-group distribution of geophagy

The 46 geophagy cases were unevenly distributed across macaque groups (Table 1, Table S1). Most occurred in the groups living on the top of the Rock (PPA, CC, and OH), while groups lower on the slope showed fewer cases (AD, RAW). We recorded one case in Lathbury Barracks (LAT) group, which regularly feeds at a garbage pit outside the reserve. Among the Northern groups, we observed one case in a Rock Gun RG adult male. Two cases occurred in a small group of sterile females (Jew’s Gate, JG) originating from Cable Car group. No geophagy was observed in Middle Hill MH, the only group without tourist contact or access to human food.

**Table 1:** Summary of observation time, number of geophagy events, geophagy rates, proportion of feeding time spent in geophagy, and terra rossa soil density across the population.

Study group	Total observation time (h)	Geophagy (number of events)*	Geophagy rate (per hour; per week)	Geophagy (percentage of feeding time)**	Geophagy (average terra rossa density value)^†^
Apes Den AD	148.1	2 (+ 1)	0.0134; 2.25	0	53
Royal Anglian Way RAW	153.9	5	0.0324; 5.443	0.56	26
Cable Car CC	107.4	10	0.0931; 15.640	1.36	58
Prince Philip Arch PPA	97.06	15	0.1543; 25.922	0.89	20
Ohara OH	34.6	8 (+ 1)	0.2313; 35.858	2.36	23
Middle Hill MH	14.6	0	0; 0	0	94
Rock Gun RG	23.8	1	0.0419; 7.039	2.5	96
Jew’s gate JG	16.3	3	0.1227; 20.613	2.77	38
Lathbury Barracks LAT	16.2	1	0.061; 10.248	0	100
Total population	612.05	44 (+ 2)	0.0718; 12.062	0.82	37

* Number of observations between August 2022 and January 2024, numbers in brackets are additional observations in April 2024; **based only on instantaneous focal follows; †Kernel values from the terra rossa density across the landscape, with smaller values representing a higher terra rossa outcrops density.

To reflect geophagy rates, we related the number of cases to each group’s observation time. During the study period, we accumulated 612 h of observation. Two events recorded in April 2024 fell outside of standardized observation sessions, so rates are based on 44 events, yielding an overall rate of 0.072 events per observation hour. Table 1 shows group-specific rates, case numbers, and observation time. As with absolute numbers, groups on the top of the Rock (PPA, CC, OH) showed rates above the population average, while those on the Western slope and Northern side had lower rates.

Following Pebsworth et al.^3^, we calculated rates in events per week, considering 168 h per week, by multiplying the hourly rate by 168 (Table 1). All calculated rates fall within the “frequent” category (> 2 per week) and far exceed this threshold. We therefore consider geophagy in Gibraltar Barbary macaques “very frequent”, with a population-level estimate of more than 12 events per week (range 0–36, Table 1), and a mean (± SD) group rate of 14 (± 12) events per week.

### Distribution across reproductive states, sex- and age-classes

Twelve geophagy events were performed by adult males (26.1% of events, including two by the same individual), 27 by adult females (58.7%, two by the same individual), four by unique subadult females (8.7%), two by unique juvenile males (4.34%), and one by infant male (2.17%) – Table S2. We could identify 24 individuals practicing geophagy, based on facial characteristics, among the 31 cases involving adult and subadult females. At the time of soil ingestion, four were nulliparous (16.7%), four post-reproductive or sterile (16.7%) - based on their age, past sterilization by the management, and evidence of reproductive termination^33^. Three females were likely pregnant based on event dates and subsequent births (12.5%), seven possibly pregnant or in non-fertile period (with no evidence of later birth or abortion, and non-maximally tumescent sexual swellings; 29.1%), two lactating (8.3%), and four neither pregnant nor lactating (16.7%).

The sex-ratio of geophagy - defined as the number of unique adult and subadult males observed ingesting soil divided by the number of unique adult and subadult females – was 11/30 = 0.366. This is slightly lower than the overall sex ratio of the studied population across sampled groups (47/105 = 0.447), suggesting a female bias in this behavior.

### Seasonality and timing of geophagy

Events were unevenly distributed across seasons: 39.1% occurred in winter, 56.5% in summer, and 4.3% in spring. We exclude the few cases observed in spring in the following comparisons, due to low sample size and lack of focal follows. Geophagy was more frequent in summer than winter, a pattern also reflected in population-level rates: 0.0935 events per observation hour in summer (0.0515 in 2022; 0.1160 in 2023) versus 0.0587 in winter (0.0357 in 2022–2023; 0.0860 in 2023–2024).

Events took place on average at 14h42 ± 3h15, ranging from 9h52 (3 Sep 2022) to 20h19 (16 Aug 2023). By season, events took place on average at 13h29 ± 1h25 in spring, 15h22 ± 3:38 in summer, and 13h52 ± 2h37 in winter. Event timing within days did not significantly differ among seasons (Welch one-way ANOVA F = 1.472, df = 2, *p* = 0.3369), across groups (ANOVA F = 1.142, df = 7, *p* = 0.358), among age-sex classes (Kruskall-Wallis Chisq = 3.102, df = 4, *p* = 0.54), or between sexes (Kruskall-Wallis Chisq = 1.543, df = 1, *p* = 0.214). Distributions across field seasons, seasons, and times of day are shown in Figs. S1–S3.

### Anthropogenic context of geophagy events

We examined whether geophagy was preceded by ingesting tourist-derived food, restricting the analysis to individual focal follows with detailed feeding records in which the target individual engaged in geophagy (*N* = 20). Because some events occurred early in follows, prior food intake could not always be confirmed. However, in 3 of the 20 cases (15%), geophagy directly followed consumption of biscuits (48 min earlier; 26 August 2023, adult female CC), dairy ice cream (7 min earlier; 29 August 2023, adult male RAW), and bread (6 min earlier; 12 September 2023, adult male RG).

### Determinants of tourist-derived food ingestion

We make the distinction between tourist-derived food (any food item provided or taken from tourists and visitors), and non-transformed food (those provided by the macaque management including fruits, vegetables and seeds listed in^25^, and naturally foraged food items). At the population level, focal follows showed that 80.4% of feeding time was devoted to non-transformed food, 18.8% to tourist-derived food, and 0.8% to geophagy (Fig. S5), corresponding to 11.16%, 2.6%, and 0.11% of total observation time, respectively.

Given the distinctive human-primate interactions in Gibraltar, with high-calorie, high-fat, and dairy-rich foods frequently provided by tourists, we hypothesized that geophagy in this population could be a dietary response to anthropogenic pressure and diet. We therefore examined the determinants of tourist-derived food ingestion across the population using a binomial GLMM assessing the likelihood of ingesting tourist-derived food, and a zero-inflated negative binomial GLMM modelling the number of tourist-derived food ingestion events per focal follow. In both models, we included as fixed effects anthropogenic pressure, non-transformed food feeding time, sex (female, male) and season (winter, summer), while setting group and dates as random effects.

**Fig. 2:**
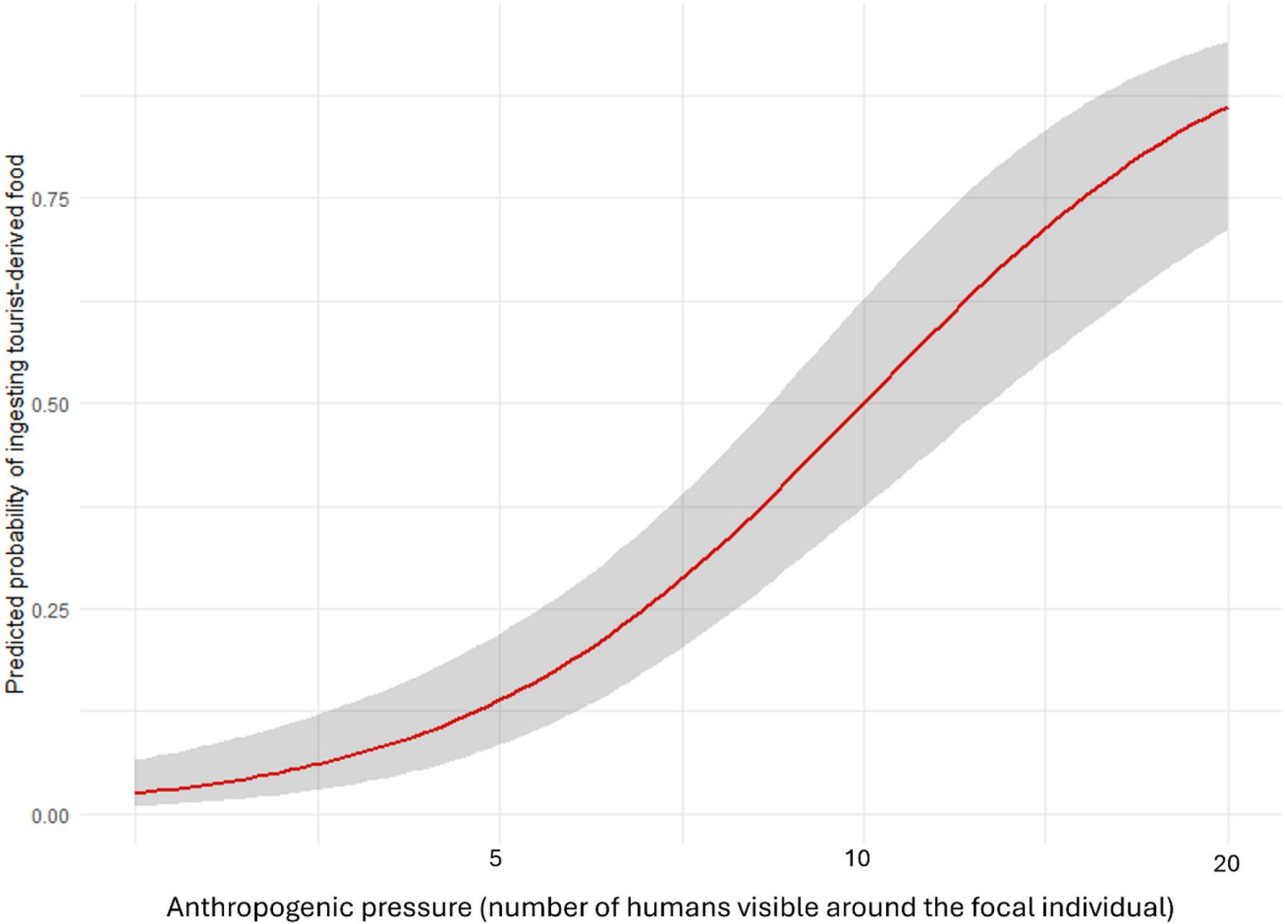
Effect of anthropogenic pressure on the probability of ingesting tourist-derived food. Model line is depicted in red, with the shaded area representing the 95% confidence intervals.

The test predictors significantly influenced the likelihood of ingesting tourist-derived food (full-null model comparison: likelihood Ratio Test (LRT): χ ² = 66.34, df = 4, P = < 0.001, Table S3). Macaques were more likely to consume tourist-derived food under high anthropogenic pressure (estimate ± SE = 0.914 ± 0.144, df = 1, *P* < 0.001; Table S3, Fig. 2), and less likely in winter than in summer (estimate ± SE = – 0.514 ± 0.259, df = 1, *P* = 0.047; Table S3). Neither sex nor time spent feeding on non-transformed food had a significant effect (Table S3) on the tourist-derived food consumed. Although a binomial GLM indicated a “group” effect (Likelihood Ratio Test (LRT): χ ² = -18.944, df = 7, *P* = 0.008), *post-hoc* Tukey tests found only Apes Den AD significantly less likely than Prince Philip Arch PPA to ingest tourist-derived food (AD-PPA: estimate ± SE = -1.229 ± 0.382, z = -3.220, *P* = 0.028).

The amount of tourist-derived food ingested was also significantly predicted by the test predictors (full-null model comparison, LRT: χ ² = 46.62, df = 4, P = < 0.001, Table S4). Consumption increased with anthropogenic pressure (estimate ± SE = 0.946 ± 0.161, df = 1, *P* < 0.001; Table S4, Fig. S6) but decreased with more time spent on non-transformed food (estimate ± SE = -0.302 ± 0.131, df = 1, *P* = 0.021; Table S4, Fig. S7). Ingestion was lower in winter than summer (estimate ± SE = -0.563 ± 0.246, df = 1, *P* = 0.022; Table S4). No sex effect was detected (Table S4), and no significant “group” effect on the ingested amount was found (LRT: χ ² = 12.43, df = 7, *P* = 0.087). Patterns of tourist-derived food ingestion broadly tracked anthropogenic pressure across groups and seasons (Figs. S8–S10).

### Spatial distribution of geophagy events and clay outcrops

A survey of the locations of all visible terra rossa (red soil) outcrops along accessible roads and trails across the study site (Fig. S4) recorded a total of 184 outcrops, used to generate a soil availability distribution via kernel density utilization distribution (UD) estimates (Fig. S4).

Terra rossa outcrops are unevenly distributed across the reserve (Fig.S4), mainly concentrated in the highest central areas along the upper road crossing the home ranges of O’Hara Battery OH, Prince Philip Arch PPA and Cable Car CC groups. The Northern and Southern sides had much lower outcrop densities. Apes Den AD group ranges lower on the western slope, where outcrops are sparse. Most geophagy events occurred in areas of high outcrop density, with an average kernel value of 37 ± 24 (range: 9–100; lower values indicate higher densities). Group-level differences in geophagy rates reflected spatial variation in outcrop density, with OH and PPA groups showing the highest frequencies in areas of particularly high outcrop density (Table 1).

### Determinants of geophagy

We fitted a binomial GLMM to the focal follow dataset to assess whether anthropogenic pressure — via tourist-derived food ingestion — and terra rossa outcrop density (measured as utilization distribution kernel values of outcrops allocated to locations used by the macaques during follows) affected the likelihood of geophagy. Fixed effects were thus anthropogenic pressure, non-transformed food feeding time, tourist-derived food feeding time, terra rossa outcrop density, sex, and season; group and dates were random effects. Within focal follows, geophagy by the focal individual was rare (20 cases; 5.2% of focal follows). None of the test predictors significantly affected the likelihood of observing geophagy during focal follows (full-null model comparison, LRT: χ ² = 8.10, df = 6, *P* = 0.23). A “group” effect was detected (LRT: χ ² = 19.067, df = 7, *P* = 0.007), but *post-hoc* tests identified no significant pairwise differences, likely due to the low frequency of events (0 or 1 per follow).

**Fig. 3:**
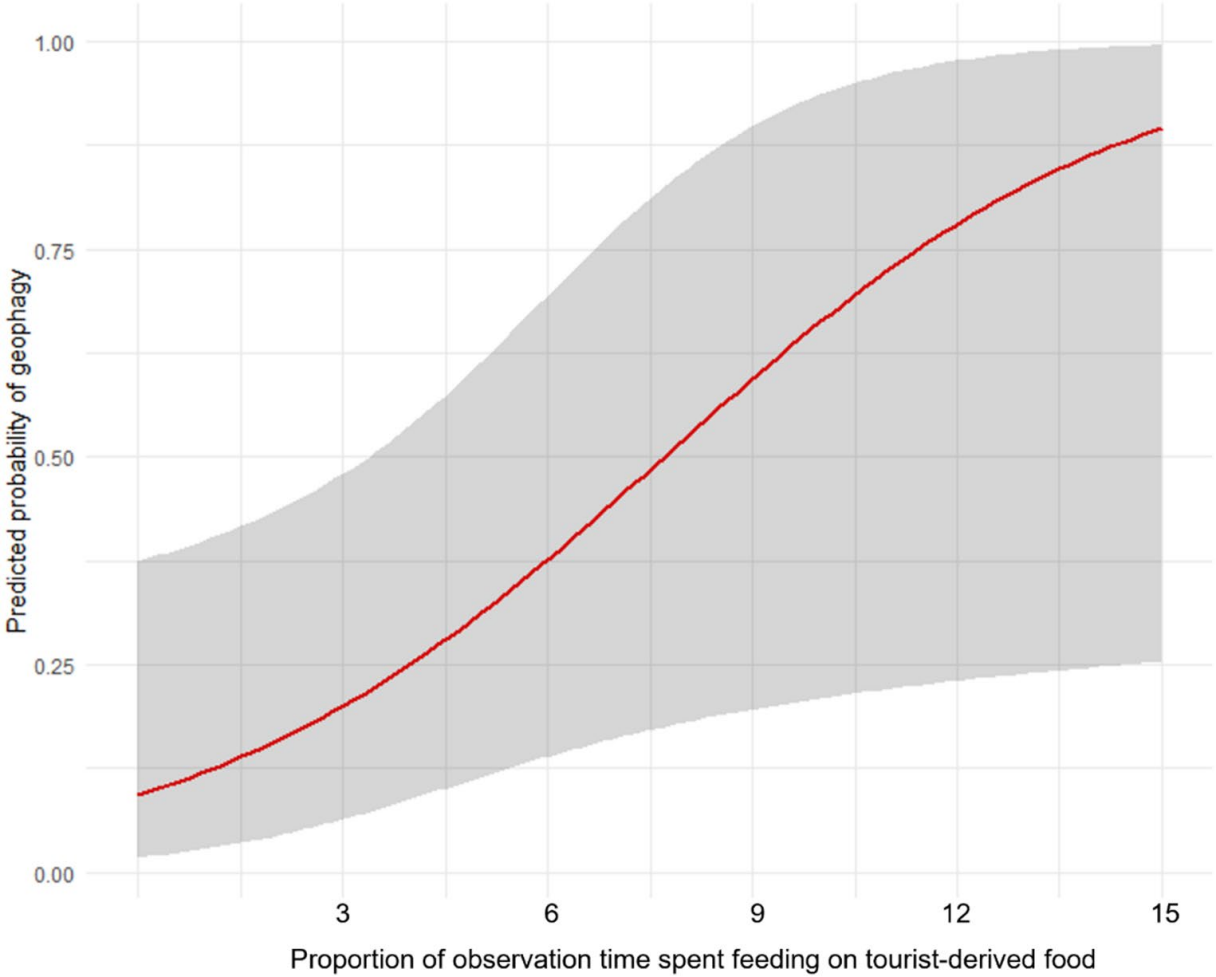
Effect of the proportion of observation time spent feeding on tourist-derived food on the probability to observe geophagy, across all groups and seasons. Model line is depicted in red, with the shaded area representing the 95% confidence intervals.

Because most geophagy events occurred outside focal follows, we conducted a second analysis at the seasonal scale, where test predictors significantly influenced the likelihood of geophagy (full-null model comparison, LRT: χ ² = 12.01, df = 6, *P* = 0.061, Table 2). Geophagy was more likely when more tourist-derived food was ingested (estimate ± SE = 0.884 ± 0.431, df = 1, *P* = 0.040; Table 2; Fig. 3), while other predictors were not significant (Table 2). No group effect was detected (LRT: χ ² = 11.11, df = 7, *P* = 0.133). Model fit was modest (marginal R² = 0.274).

**Table 2:** Determinants of the likelihood to perform geophagy.

Terms	Estimate	SE	Z	95% CI	*P*
Intercept	– 0.235	0.586	– 0.402	– 1.446; 0.902	NA
Anthropogenic pressure^*, †^	0.188	0.438	0.66	– 0.729; 1.097	0.463
Tourist-derived food feeding time^*, †^	0.884	0.431	2.049	0.096; 1.855	**0.040**
Non-transformed food feeding time^*, †^	0.668	0.418	1.596	– 0.089; 1.588	0.110
Terra rossa outcrop density^*, †^	– 0.299	0.375	– 0.798	– 1.132; 0.417	0.425
Sex – male^*, ‡^	– 1.153	0.741	– 1.556	– 2.715; 0.238	0.119
Season – winter^*, §^	0.894	0.726	1.230	– 0.486; 2.408	0.218

Results from a binomial GLMM from a seasonal dataset per group and sex (*N* = 47).

Marginal effect sizes (R²), counting for the variance explained by fixed effects, was 0.274, while conditional R2, counting for the variance of both fixed and random effects, was 0.274; * test predictor; † z-transformed; ‡ reference level is female; § reference level is summer; p-values in bold indicate a statistically significant effect (*p* < 0.05). Maximum VIF: 1.39.

Seasonally, a zero-inflated negative binomial GLMM, with observation time as an offset term, revealed that the number of geophagy events was significantly affected by the test predictors (full-null model comparison, LRT: χ ² = 19.18, df = 6, *P* = 0.0003, Table 3). More events occurred in areas with higher terra rossa outcrop’s density (estimate ± SE = -0.625 ± 0.275, df = 1, *P* = 0.023; Table 3), males performed fewer events than females (estimate ± SE = -1.909 ± 0.421, df = 1, *P* < 0.001; Table 3), and fewer events occurred in winter than summer (estimate ± SE = -1.245 ± 0.360, df = 1, *P* < 0.001; Table 3). Anthropogenic pressure and feeding time (non-transformed or tourist-derived) were not significant. No group-level differences were detected (LRT: χ² = 13.43, df = 7, *p* = 0.062). Model fit was high (marginal R² = 0.619).

**Table 3:** Determinants of the number of geophagy events.

Terms	Conditional model (log-odds of success)	SE	Z	95% CI	*P*	Zero-inflation model (log-odds of excess of zeros)	SE	Z
Intercept	– 4.048	0.317	– 12.761	– 4.670; – 3.427	NA	– 0.444	0.392	– 1.133
Anthropogenic pressure^*, †^	– 0.097	0.178	– 0.545	– 0.447; 0.252	0.585			
Tourist-derived food feeding time^*, †^	0.294	0.187	1.572	– 0.072; 0.660	0.115			
Non-transformed food feeding time^*, †^	0.068	0.223	0.308	– 0.368; 0.506	0.758			
Terra rossa outcrop’s density^*, †^	– 0.625	0.275	– 2.269	– 1.165; – 0.085	**0.023**			
Sex – male^*, ‡^	– 1.909	0.421	– 4.527	– 2.735; – 1.082	**< 0.001**			
Season – winter^*, §^	– 1.245	0.360	– 3.450	– 1.952; – 0.537	**< 0.001**			

Results from a zero-inflated negative binomial GLMM.

Marginal effect sizes (R²), counting for the variance explained by fixed effects, was 0.619, while conditional R2, counting for the variance of both fixed and random effects, was 0.619; * test predictor; † z-transformed; ‡ reference level is female; § reference level is summer; p-values in bold indicate a statistically significant effect (*p* < 0.05). Maximum VIF: 1.38.

### Social context of geophagy events

We documented the social contexts of geophagy events. Most (69.6%, *n* = 32) were performed alone (a single individual ingesting soil at a particular outcrop), while 30.4% (*n* = 14) involved multiple individuals co-feeding at the same outcrop or taking turn ingesting soil at the same outcrop. In two cases, competition around small outcrops of which access can easily be monopolized was evident, with adult females displacing others, which may explain the low frequency of collective geophagy. Social geophagy is illustrated in Movies S3–S4, where an adult male consumes clay (Movie S3) followed by an adult female at the same outcrop (Movie S4). Identities of individuals present in the vicinity of those practicing geophagy was however not systematically recorded. Detailed account of such social setting will be necessary to quantify the opportunities of socially learned this behavior, in particular by infants and juveniles.

Although rather practiced solitarily, 89.1% of cases (*n* = 41) occurred in the presence of conspecifics as visible bystanders (within 20 m), while it only rarely occurred completely alone without any visible bystander within 20 m (10.9%, *n* = 5). On average, 4.25 ± 3.46 individuals (range: 0–16) were present as bystanders within 20 m. Thus, geophagy in Gibraltar is embedded in a social setting, offering opportunities for observation and social learning. Movie S5 illustrates a juvenile observing an adult female ingesting terra rossa soil.

### Soil presentation experiments

**Fig. 4:**
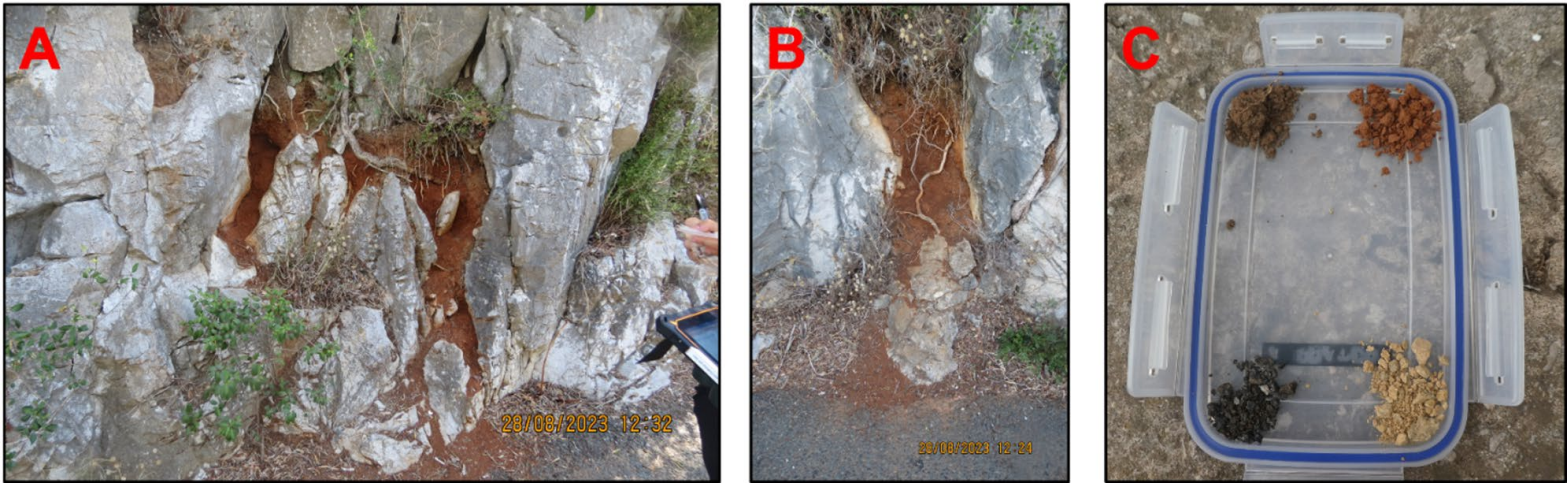
Types of soil sampled in the Upper Rock Nature Reserve of Gibraltar. (**A**–**B**) Examples of red clay outcrops emerging from the rock wall, along concrete roads; (**C**) protocol used in soil presentation experiments, including the four types of soils found in Gibraltar – upper left: black earth; upper right: red soil; bottom left: tar; bottom right: yellow soil.

We conducted 124 soil presentation experiments to 108 distinct individuals across seven groups (Table S5) to assess local soil preferences, presenting on a plastic tray the four types of Gibraltar soil simultaneously to individual macaques (Fig. 4). The samples presented were all collected from the same locations for each respective soil type, to ensure equal presentation context and material across experiments. The soil positions were randomized for each trial and controlled in subsequent analyses. Each trial consisted of a presentation of the set-up to a single individual macaque. Middle Hill and Lathbury Barracks groups were not tested due to access difficulties. We tested 29 subadult/adult males (23.4%), 54 subadult/adult females (43.5%), 35 juveniles (28.2%), and six infants (4.8%) (Table S5).

Among the 124 presentations, macaques interacted with soil samples in 33.8% of them (*n* = 42), with rates of interaction with the set-up varying by group (0% − 48.1%). Juveniles (74.2%) and infants (83.3%) were the most responsive, while adult males (3.4%) and females (18.5%) showed low engagement (Table S5), hereby likely reflecting the stronger exploratory and manipulative interest of juveniles compared to adults. The presentation of soil on a plastic tray may have affected the willingness and/or curiosity of tested individuals, as it differs from naturally occurring soil outcrops. However, the lack of interaction by adults was less characterized by signs of fear or neophobia than by disinterest, the individuals preferring to carry on their activities or being focused on tourists and/or conspecifics. We are thus confident that the experimental set-up did not influence the propensity of individuals to interact with its content.

We recorded first-choice interactions in 39 presentations out of the 42 in which individuals interacted with the protocol. In 3 cases out of 42 in which they interacted with the protocol, we could not obtain information on first choice due to visibility issues (tourists obstructing the camera). Among the 39 trials in which we obtained information on first choice, terra rossa was most commonly chosen (51.3%), followed by tar (25.6%), black earth (15.4%), and yellow soil (7.7%), broadly reflecting natural observed geophagy patterns. Of the 42 interaction trials, we could however assess ingestion patterns, with 21 (50%) resulting in ingestion: tar (10 cases, 23.8%), terra rossa (5 cases, 11.9%), black earth (3 cases, 7.1%), and yellow soil (3 cases, 7.1%).

Restricting analyses to AD, RAW, and CC (groups with more than 20 trials and interaction rates > 30% - Table S5), no group effect was found for first-choice interactions or terra rossa ingestion. However, tar ingestion differed significantly between groups (χ² = 7.001, df = 2, *p* = 0.030, Table S6), being more common in Apes Den AD than in Cable Car CC (estimate ± SE = − 2.356 ± 1.164, *p* = 0.043) or Royal Anglian Way RAW (Table S6). Apes Den AD accounted for 70% (7 of 10) of tar ingestion, while terra rossa ingestion was more evenly distributed across groups.

These results suggest that terra rossa is the overall preferred soil type, but Apes Den shows a distinctive tendency to ingest tar compared to other groups.

### Variation in geophagy across Barbary macaque populations

**Fig. 5:**
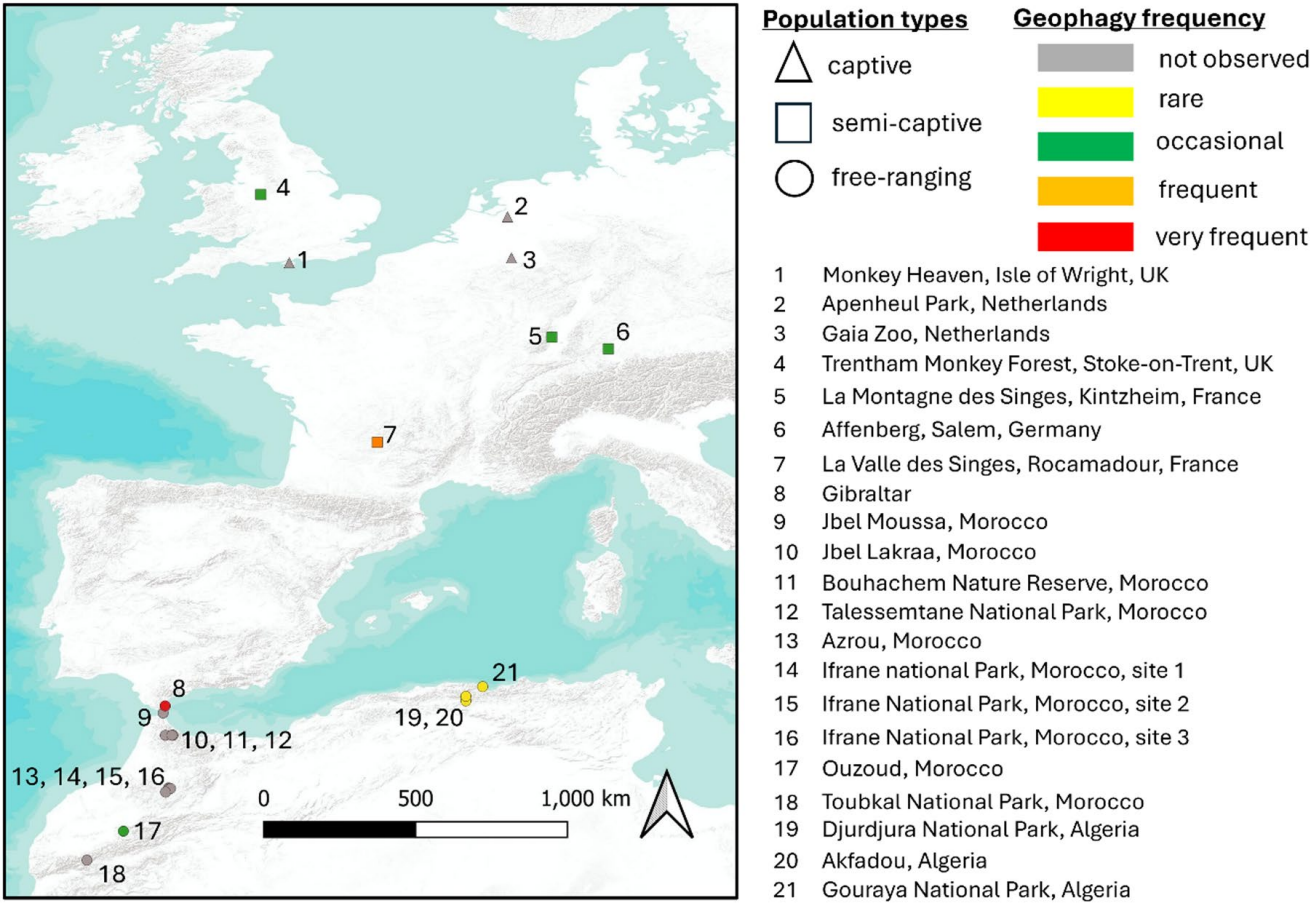
Frequency of Barbary macaque geophagy across sites, based on anecdotal reports by contacted researchers and following the categories proposed by Pebsworth et al., 2019. The sites correspond to those from which contacted researchers provided information on observed geophagy and from which precise geographic locations could be retrieved. The shape of symbols corresponds to the type of population; the color coding corresponds to reported geophagy frequencies; the numbers refer to the different study sites. This map was created using QGIS Desktop 3.38.3 Grenoble https://www.qgis.org.

We identified researchers and study sites working on Barbary macaques from the literature, and contacted corresponding authors to ask whether they had ever observed geophagy at their sites and related information regarding its practice. Geophagy was reported in 9 of 26 study sites (34.6%) we successfully contacted, including all four European semi-captive and five wild populations (Fig. 5). None of the reports we obtained had quantified rates of geophagy, so we extrapolated the information provided by researchers into the broad categories proposed by^3^(rare, occasional, frequent, very frequent).

In semi-captive populations, geophagy was typically occasional and seasonal. At Affenberg, Salem, Germany, it occurred mainly among juveniles and subadults (R. Hilgartner, S. Preuschoft, N. Mueller-Klein, pers. comm.). Occasional cases were reported at La Montagne des Singes, Kintzheim, France (R. Hilgartner, pers. comm.) and Monkey Forest, Trentham-at-Stoke, UK (R. Hilgartner, M. Lovatt, pers. comm.). In La Vallée des Singes, Rocamadour, France, it was observed more regularly (R. Hilgartner, J. Fisher, pers. comm.).

In free-ranging populations, geophagy was rarer, occasionally observed in a tourist-fed group in Ouzoud, Morocco (A. El Alami, pers. comm.), and rarely in adults of an anthropized group at Gouraya National Park, Algeria, where charcoal ingestion was also noted (M. Boumenir, N. Menard, pers. comm.). Possible additional cases occurred in Djurdjura National Park, Akfadou, Bejaia, Algeria (N. Menard, pers. comm.). In Gibraltar, geophagy occurred occasionally in Middle Hill group during 2003–2005, when individuals were fed by tourists and military personnel (Dana Pfefferle, pers. comm). Occasional rock licking and charcoal ingestion were also reported in Ifrane National Park, Morocco (B. Majolo, pers. comm.).

Overall, these findings indicate that geophagy in Barbary macaques is not unique to Gibraltar but varies widely across sites, often being occasional, seasonal, or age-class specific. The Gibraltar population stands out for its comparatively high frequency, observed across multiple age-sex classes, making geophagy distinct from most other studied populations.

## Discussion

This study provides the first detailed account of geophagy in Barbary macaques, particularly in Gibraltar, underscoring the role of human-primate interfaces in shaping behavioral adaptations. Geophagy in this species had previously been mentioned by Pebsworth et al.^3^ who cited Rowe and Meyers^34^, themselves referring to Drucker (1984)^35^, but beyond these anecdotal reports, no systematic study of this behavior had been undertaken so far, in Gibraltar or any other Barbary macaque population. The inconspicuous aspect of geophagy in Gibraltar, and lack of systematic long-term study, may have impaired the study of such behavior in this population. Geophagy in the Gibraltar macaque population is discussed in light of the supplementation and protection hypotheses within an anthropogenic context. We also consider the role of social learning and cultural transmission to explain this behavior.

Geophagy was unevenly distributed across the study area and among groups. The highest frequencies occurred in groups on the upper and central part of the Rock, where red soil (terra rossa) outcrops are common and anthropogenic pressure — tourist presence and human–macaque interactions — is greatest. In contrast, groups on the lower western slope showed fewer events for an equivalent observation time. Middle Hill, the only group without tourist contact and no longer consuming human food showed none, but this could be due to lower observation time than in the touristic groups. Reported anecdotal observations of Middle Hill consuming soil in the past, when they regularly interacted with military personal and tourists on the Eastern side, indicate that this behavior may have been lost, or is still practiced but at much lower rates than in the past. This spatial pattern indicates that geophagy in Gibraltar is influenced by both ecological availability of soil resources (especially terra rossa outcrops) and the anthropogenic context.

Compared to non-primate animals known for practicing geophagy, the population rate in Gibraltar macaques of 12 events per week can be considered as highly frequent. For example, in a population-level investigation of salt lick visitation in Amazonian large-bodied mammals, using camera-traps, it was found that agouti, paca, collared peccary, Brazilian porcupine and tapirs visited salt licks between 24.2 and 15.4 times per 100 camera nights^12^, corresponding to 1.7 to 1.08 times per week. Interestingly, the same study found that red howler monkeys were the most selective species with a mean frequency visit of 8.2 visits per 100 camera nights^12^. The Gibraltar geophagy rates remain however much lower than extreme rates observed in snowshoe hares in Alaska that visit salt licks nightly between 3 and 9 times per night^36^.

Compared to other primates in which geophagy rates have been classified as frequent^3^ and that provide quantitative information, the population-level 12 events per week in Gibraltar macaques are comparable to the highest reported rates, including the 16.4 cases per week in Ring-tailed lemurs^37^, the 11.7 cases per week in Ryland Bald-faced saki monkeys^38^ and the 14 times per week in East-African chimpanzees^39^. The geophagy rates in Gibraltar macaques are also among the highest reported in the *Macaca* genus^3^. Provisioned hybrid macaques in Hong Kong showed up to 33.9 events per week^40^– one the highest frequency so far reported in mammals - Formosan macaques about 11.7 per week^41^, and Japanese macaques about 8.8 per week^42^ whereas wild Bornean pig-tailed macaques visited salt licks only 0.3 times per week^43^. With more than 12 events per week on average, Gibraltar macaques fall in the high end of this range. Such frequent geophagy is to be related with other provisioned or insular macaque populations^40,41^ where limited dietary diversity and high exposure to anthropogenic foods may increase the needs for mineral supplementation or detoxification. Further comparative work, along anthropogenic gradients, would prove useful in unraveling these relationships.

Higher female rates of geophagy than what would be expected from the demographic population sex ratio seems to indicate a female bias in geophagy in the Gibraltar population. This is similar to what has been reported, among non-human primates, in Northern muriquis^44^, in chacma baboons^45^, in Bornean orangutans^46^, in Milne-Edward’s sifaka^47^ and in Golden snub-nosed monkeys^48^. Within *Macaca* genus, this contrasts with Tibetan macaques (*M. thibetana*), which show no sex difference in geophagy^49^, but aligns with higher frequencies in female rhesus macaques (*M. mulatta*)^7^, and in female Japanese macaques^42^. Formosan macaques (*M. cyclopis*) also show higher geophagy frequencies in females, especially during the breeding season^41^. In Gibraltar, however, geophagy did not increase in winter, the species’ breeding season, and occurred across all female reproductive states (nulliparous, pregnant, lactating, post-reproductive). Thus, while the supplementation hypothesis predicts increased geophagy to meet reproductive mineral demands, especially during pregnancy and lactation, our results provide little support for it. However, this descriptive sex difference based on sex-ratio of geophagy compared to the population sex-ratio shall be taken with caution, as we did not find statistically significant sex differences in geophagy rates neither in ingestion of tourist-derived food in our models. Other studies in this population revealed complex site-specific peculiarities in human-macaque interactions^50^, with higher food items snatching at Prince Philip Arch (PPA, CC, OH groups) for males, but higher at Saint Mickael’s Cave (RAW group) for females. Similarly, even if non-significant, signs of effects in our models show that males are less likely than females to ingest tourist-derived food (Table S3), but that males ingest larger quantities of tourist-derived food than females (Table S4). Site-specific rather than group specific investigation of the relationship between anthropogenic pressure, access to tourist-derived food and geophagy, across sexes and eventually dominance hierarchy, would be critical in addressing these differences.

Ingestion of soil for supplementation of diet cannot be fully ruled out. First, the hypothesis predicts that if tourist-derived food lack of essential nutrients, more geophagy is expected when more tourist-derived food is ingested, a pattern confirmed by our analyses. Second, wild Barbary macaques have a flexible diet^51–53^, yet Gibraltar macaques show reduced dietary diversity. In North Africa, they consume acorns, lichens, mushrooms, and invertebrates^51,54,55^, items largely missing in Gibraltar (especially arthropods), where macaques rely mainly on provisioned food with limited wild plant matter. Provisioning of non-transformed food also varies: some groups (notably PPA and OH) receive mostly seeds and monkey nuts, lacking fresh fruits and vegetables. Here, geophagy might serve as a nutritional supplement due to low levels of insectivory, or even as a “hunger-cutter,” offsetting deficiencies in provisioned diet. Mineralogical and chemical analyses of soils, including analysis of clay content known for its toxin adsorption properties, paired with nutritional profiling of non-transformed foods, will be key to assessing this possibility in the future and fully understand which minerals and components the macaques may benefit from. Also, given that protection and supplementation are not mutually exclusive and can apply differently at various stage of life, precise inter-sites comparisons of soil composition, texture and mineralogy, in relation to the specific age-sex class and reproductive status of individuals consuming soils at these sites would be required.

Nevertheless, seasonality in Gibraltar macaque’s geophagy suggests a different mechanism than those related to sex-specific physiological needs, or nutritional deficiencies. Rates were consistently higher in summer than winter, across sexes and age-classes. While this could reflect more lactating females in summer, our results suggest that it is more likely linked to the seasonal tourist influx. Visitor numbers peak in summer^56^, increasing both anthropogenic food availability and human–macaque encounters. In support of this prediction, our analysis confirms that more visitors raise the likelihood of macaques consuming tourist-derived food, that more geophagy events were observed when tourist-derived food ingestion was high, while it shows no relationship with non-transformed food. However, we do not find a direct effect of number of visitors around the macaques, proxied as anthropogenic pressure, on the likelihood of geophagy, but rather a direct effect of tourist-derived food ingestion. This can be due a threshold effect in the relationship between anthropogenic pressure and access to tourist-derived food (Fig. S6), where a certain number of visitors around the macaques is necessary to create enough opportunities to access to tourist-derived food (either by pilfering or by provisioning). This threshold effect may mask any direct effect between number of tourists and geophagy. However, our results generally support the view that geophagy in Gibraltar is driven primarily by anthropogenic diets rather than by reproductive demands. This supports the protection hypothesis - soil buffers the digestive system against challenging components of human food - but not the supplementation hypothesis. The association between geophagy and provisioning with high energy, low fiber, human foods causing gastric upset was part of the findings from^3^ in their review on primates geophagy. Striking comparative evidence is found in the provisioned Arashiyama Japanese macaques^42^ where two-third of their diet is composed of provisioned items making them prone to gastric upset, and who ingest at high frequencies soils rich in clay minerals, such as kaolin that can buffer gastric related upsets.

The anthropogenic diet of Gibraltar macaques fits within this plausible pathway for a protective role of geophagy. Tourist-derived foods are high in sugar, salt, fat, and dairy but low in fiber. Because non-human primates lose lactase activity after weaning^57^, dairy can cause digestive upset. Post-ingestive feedback of malaise sensation correlated with nutritional deficiencies can lead to behaviors that correct the disorder^58^, in turn reinforcing the association between malaise and subsequent intake of soil. Soil ingestion may then relieve these symptoms by adsorbing irritants, modifying gut pH, or shaping gut microbial communities. Geophagy as a remedy against gut microbiome disturbance requires further scrutiny, but comparative evidence supports this potential link: in lemurs, soil alters or supplements gut microbiota^59–61^, and diet-induced microbiome diversity changes are widespread in primates^62–65^. Long-tailed macaques show reduced microbial diversity on a Western diet compared to a Mediterranean one^62^, and sifakas exhibit shifts linked to folivory^65^. If Gibraltar macaques use soil to counter microbiome disruptions from anthropogenic foods, this would advocate for an extension of the protection hypothesis beyond toxins and plant compounds to human-derived dietary elements.

Beyond function, geophagy in Gibraltar also shows features of a cultural trait. Although intakes seem to be performed rather solitarily, likely due to inter-individual competition around small outcrops, they occurred in majority in a social context with bystanders, offering opportunities for observations and social learning by conspecifics. How the level of competition for soil relates to feeding competition patterns in this population and limits collective geophagy remains to be explored and would require detailed dominance hierarchy information within each group. Nevertheless, even if intake rates differing across groups may be related to differences in anthropogenic pressure and availability of soil, and even if post-ingestive feedback may explain the association between tourist-derived food and geophagy, soil presentation experiments showed distinct group-level preferences: red soil was generally favored, but AD displayed a notable inclination for tar. It is unlikely related to no access to red clay for tis group, given that anecdotal accounts from locals indicate observations of AD individuals ingesting terra rossa (Brian Gomilla Monkey Talk, pers, comm.), and because terra rossa outcrops are available across their range, even if in lower density than on the upper slopes. Such variation is also unlikely to arise from independent invention alone and instead suggests local traditions shaped by social learning. Group-specific feeding preferences are indicative of social learning in human^28^ and non-human primates^29–31^. Juveniles and infants watching and imitating adults support this interpretation, as do female biases consistent with philopatric, sex-biased learning^66,67^. Thus, feeding behaviors, including geophagy, may be socially transmitted within groups, paralleling other cultural traits in non-human primates^68^. Even if tar is mostly composed of organic matter, it may still contain some minerals and still constitutes a non-food item, similar to pica behavior in humans^6,9^.

Interpretating geophagy as cultural partly depends on how culture is defined. Conservative views exclude ecology-shaped behaviors^24^, whereas newer “ecology of culture” perspectives emphasize interactions between ecological conditions and social learning^32,69,70^. In Gibraltar, geophagy seems to arise both from necessity (buffering dietary disruption from anthropogenic foods) and opportunity (readily available soil outcrops). Its persistence and group-specific patterns point to cultural transmission, supported by strong female philopatry and ample chances for observation and imitation. However, long-term monitoring of this behavior across the population, including rates of adoption by infants and juveniles, and potential changes of soil preferences in migrating males, alongside network-based diffusion analyses, would be needed to fully comprehend the mechanisms of social transmission of this behavior.

More broadly, geophagy in Barbary macaques shows marked spatial variation. Our survey confirmed it is not unique to Gibraltar, occurring in semi-captive groups in Europe and free-ranging groups in North Africa, though usually infrequently. The reported rates from other sites shall be taken with caution due to a lack of systematic quantification, but the broad categories of usage we considered are informative to assess that Gibraltar seems to be distinctive for its high rates and strong link to anthropogenic contexts. Systematic studies of geophagy in other populations are advocated to quantitatively compare geophagy rates across the species range. However, this preliminary comparison suggests that geophagy may be a facultative behavior expressed more intensely under certain ecological and human-influenced conditions, while its presence across sites reflects a broader but variable species-level repertoire.

The origins of geophagy in Gibraltar remain unclear. The current population descends from Algerian and Moroccan stocks introduced after WWII^71,72^, and our survey shows that geophagy occurs - though rarely - in some North African groups, suggesting it may have been imported. Anecdotal reports from the early 2000s in the Middle Hill group also indicate that its prevalence can fluctuate or disappear. As with other primate cultural traits, geophagy may be sensitive to demographic change, environmental shifts, or anthropogenic pressure^73,74^. Long-term, multi-group monitoring will be essential to understand its emergence, spread, persistence, and potential loss.

Several limitations hinder the interpretation and generalization of our findings, including a relative small dataset, especially regarding the number of geophagy events; an unequal observation time across the groups, especially those with less interactions with humans (MH, RG and LAT), that may impact on the rates of observed geophagy; the lack of rank data for individuals when considering access to tourist-derived food, although our main interest remained on assessing the impact of anthropogenic pressure on this variable; and the lack of systematic soil survey at all the other sites we have been able to contact. We also acknowledge that experiments in wild settings cannot fully control external variables such as observing bystanders, isolation and selection of individuals, and their motivation to participate. We nevertheless find relevant and informative the outcomes of the soil presentations we undertook, although more systematic and repeated tests would contribute to consolidate their interpretations and conclusions. We also acknowledge that mineralogical and chemical analyses of the soil, across the landscape and outcrops, would be highly needed to fully comprehend the benefits obtained by the macaques, teasing apart between protective and nutrient functions, and to test the potential link with microbiome disruption.

Nevertheless, geophagy in Gibraltar Barbary macaques appears to be one of the most frequent and patterned cases reported in *Macaca* genus. Its link with anthropogenic diets suggests a protective role against digestive impacts of human food, while female bias and group-level preferences indicate social learning and cultural transmission. Rather than an isolated curiosity, Gibraltar geophagy illustrates how human–primate interfaces create new challenges, shape diet, and foster behavioral traditions. By documenting this behavior in depth, our study highlights the need to consider both ecological pressures and cultural processes in primate foraging within changing environments. Future research integrating chemical, mineralogical, microbiome, and long-term social data will clarify the physiological benefits of soil ingestion and track its transmission, persistence or loss across populations. Thus, geophagy in Gibraltar is not merely a feeding anomaly, but a functional and cultural response to an anthropogenic landscape, underscoring the adaptive flexibility of Barbary macaques and the strong influence of humans on primate behavior.

## Materials and methods

### Study area

The study population of Barbary macaques inhabits the Gibraltar Nature Reserve: Upper Rock (36°8’N, 5°21’E), established in 1993. The reserve covers 2.5–3 km from North to South along the middle and upper slopes of the Jurassic limestone “Rock of Gibraltar”, culminating at 424 m above sea level. Most infrastructures lie on the western side while the reserve contains a mosaic of high maquis, cliff faces, garrigue, fire breaks and other habitats. It is a highly anthropogenic environment, with extensive roads and trails, a cable car, restaurants, viewpoints, dedicated monkey-watching areas, and attractions such as a cave, tunnels and historic gun batteries. Several hundred thousand tourists visit the Upper Rock annually, with the macaques as the main attraction^21,22^. Visitors often approach, touch, or provision the macaques – despite regulations - which can lead to aggressive encounters^56^. Groups experience different levels of non-transformed, tourist-derived, and naturally foraged foods^25^, reflecting variation in human visitation and interaction intensity, as supported by isotopic analyses^75,76^.

### Study population

Barbary macaques are diurnal primates living in multi-male multi-female groups, characterized by female philopatry and male dispersal. As seasonal breeders, mating occurs in autumn and winter^77^. Primarily terrestrial and omnivorous, their diet comprises fruits, seeds, leaves, herbaceous vegetation, grass, tree bark, and arthropods^51,54^. In Gibraltar, tourist-derived foods are common^25^, although variable across groups, and consumption of arthropods appears scarce (SL personal observation) compared to North-African populations. Most of daily diet consists of fruits, vegetables, seeds and fresh water provided by the local management authority^25,78^,

Historically, this population experienced rapid growth phases linked to rising tourist pressure in the 1980–1990s, stabilizing at around 230 individuals since the 2000s, currently distributed across eight stable groups (Apes Den group AD, Middle Hill group MH, Royal Anglian Way group RAW, Prince Philip Arch group PPA, Cable Car group CC, O’Hara Battery group OH, Rock Gun group RG (also called Farrington’s Barracks), Lathbury Barracks group LAT (also called Incinerator group)^21,71^.

Demographic parameters for the study groups are provided in Table S7. Their current home ranges, based on cumulative locations from focal follows recorded with GPS devices carried by observers, are illustrated in Fig. 1 as 95% kernel density utilization distributions. These groups experience varying levels of tourist pressure and human interactions. Major interaction sites (Fig. S11) include Apes Den, St Michael’s cave (used by RAW group), Prince Phillip’s Arch (used by CC, PPA, OH and AD groups) and the cable car station (used by CC group). RG group, which split from MH group in the early 1990s^79^, also regularly encounters tourists near the Princess Caroline Battery and the Great Siege Tunnels at the northern tip of the reserve.

### Soil types and geological setting

Terra rossa^80^ is the predominant soil type in the Upper Rock. These firm, seemingly clay-rich deposits form through chemical weathering of Jurassic limestone, where soluble carbonates are leached out, leaving insoluble residues of clay minerals and iron oxides. Hematite gives the soil its characteristic red color, though local soils can range from deep red to brown and, in some pockets, dark gray or black depending on organic matter content. Terra rossa is widespread across the reserve’s karst slopes and is the main material ingested during macaque geophagy.

In this study, we refer to terra rossa exposures as “terra rossa outcrops”. These are surface exposures of red soil created where erosion, weathering, or disturbance has removed overlying layers. On the Upper Rock, they commonly occur between exposed rocks or along roadside cuttings (Fig. 4), and can be of variable size, some being small enough for a single macaque individual to monopolize it and prevent access to other individuals. Their distinctive color and texture make them easy to identify during surveys. Other soils considered include a goethite-rich variant of Terra rossa (yellow soil) formed through soil eluviation^80^; Mediterranean humus (black earth) rich in organic matter^81^, and tar from asphalt road. Tar is mostly composed of organic matter but may also contain a certain amount of minerals, so we consider it as non-food items such as soil, and thus includes tar ingestion into geophagic behaviors.

### Data collection

We conducted observations over four field seasons: summer #1 (19 August − 6 September 2022), winter #1 (13 December 2022–6 January 2023), summer #2 (15 August 2023–14 September 2023), and winter #2 (6–18 December 2023 - and 6–17 January 2024). An additional short field period took place from 7 to 12 April 2024. Inter-observer tests between observers were conducted each season and revealed consistency across observers (Kappa score 0.85). Observation effort was not evenly distributed across all groups, as monitoring of OH, RG, MH and LAT began only in winter #1, and MH, RG, JG and LAT were generally followed less than the other groups. For statistical analyses, we combined data into two seasons, winter (winter #1 and #2) and summer (summer #1 and #2).

We collected behavioral data on handheld devices using Cybertracker (www.cybertrackeronline.com), combining one-hour individual focal follows with group scan sampling^82^. Opportunistic *ad-libitum* sampling recorded geophagy observed outside focal or scan sessions. Whenever geophagy occurred, we recorded the individual’s identity, age/sex class, GPS location, and filmed the event when possible. We provide a description of ingestion patterns in the result section. We calculated geophagy rates (Table 1) for each group and season as the number of confirmed events divided by total observation time.

Age classes followed established Barbary macaques definitions^83–85^: adult males (> 7 years), subadult males (5–7), juvenile males (1–5), adult females (> 5), subadult females (3–5), juvenile females (1–3), and infants (< 1 year).

During instantaneous focal follows of one-minute intervals on adult individuals, we recorded the number of humans within 3 m and 20 m of the focal individual, ongoing activities including interactions with humans, social interactions with other macaques (although exact identities of macaques interacting with the focal one was only possible after this study period), and the number of visible in-group conspecific. GPS locations were logged every 30 s using handheld Garmin© GPS units. Group scans between focal follows recorded the location, activity including human-macaque and social interactions, identity and age/sex class of each visible individual.

Observation ran from 09:00 to 18:00 in winter and until 21:00 in summer, alternating group order. Total observation time per group is shown in Table 1. We excluded April 2024 from quantitative analyses, as it lacked focal follows, but qualitatively report notable geophagy events for this month (numbers in brackets: geophagy events, Table 1).

From 379 focal follows (mean ± SD data points per follow: 46.09 ± 21.09; total: 17470) collected between August 2022 and January 2024, we extracted:

- non-transformed food feeding time (mean ± SD = 0.114 ± 0.173; range 0–0.872): proportion of observation time spent feeding on provisioned (by the local management at feeding platforms) and naturally foraged food. Non-transformed foods include fruits, vegetables and seeds listed in O’Leary^25^. Several feeding stations within the reserve differ in food type, provisioning frequency, and group access.
- tourist-derived food feeding time (mean ± SD = 0.026 ± 0.062; range 0–0.46): proportion of observation time spent feeding on foods obtained from visitors, including chocolate bars, cereal bars, crisps, sweets, bread, salted seeds, bananas, dry pasta, sodas, biscuits, and ice-cream^25^.
- number of tourist-derived food ingestion events per focal follow (mean ± SD = 1.20 ± 2.90; range 0–24).
- anthropogenic pressure (mean ± SD = 5.139 ± 6.208; range 0–35.64): mean number of humans visible within 20 m of the focal individual.

We calculated feeding-time proportions relative to the total observation time rather than the total feeding time to reduce collinearity between non-transformed and tourist-derived feeding variables. Time spent in geophagy was not included in the variables non-transformed food feeding time and tourist-derived food feeding time.

We also constructed a seasonal dataset: for each group, season (summer #1, winter #1, summer #2, winter #2) and sex, we compiled the number of geophagy events, whether geophagy occurred (yes/no), the mean anthropogenic pressure (mean ± SD = 4.777 ± 3.573), the proportions of time spent feeding on tourist-derived food (0.025 ± 0.031) and non-transformed food (0.116 ± 0.082), and the mean soil availability (48.62 ± 24).

### Spatial analysis

We surveyed and recorded the GPS locations of all visible terra rossa outcrops along accessible roads and trails across the Upper Rock Nature Reserve (Fig. S4), covering 12.3 km. Most consisted of easily detectable red soil. In total, 184 outcrops were recorded and used to generate a soil availability distribution via kernel density utilization distribution (UD) estimates^86,87^ (Fig. S4). Kernel values ranged from 4 to 100, with lower values indicating higher spatial density. Each geophagy event was assigned a terra rossa spatial density value based on its position in this distribution. We applied the same procedure to focal follows with GPS data by calculating the mean terra rossa spatial density value across all locations for each follow. This mean value served as the terra rossa outcrop density variable in our models (mean ± SD = 42.470 ± 20.696; range 9–100).

We performed spatial analyzes in R (v.4.4.1) using the packages ‘*adehabitatMA*’(v.0.2-6)^87^, ‘*sp*’ (v.2.2-0)^88^, ‘*rgdal*’ (v.1.6-4)^89^ and ‘*sf*’ (v.1.0–21)^88^. Soil-location utilization distributions were computed with the “kernelUD” function in ‘*splancs*’ (v.2.01-45)^90^.

### Soil presentation experiments

To assess group-specific soil preferences, we conducted soil presentation experiments in seven groups. Four soil types found in Gibraltar (Fig. 4C) we presented simultaneously to separate individuals:

1. terra rossa – commonly consumed;
2. tar – observed consumed only in Apes Den group;
3. yellow soil – observed consumed in three events;
4. black soil – observed consumed in three events.

Experiments took place in summer 2023 and between January-March 2025. The samples presented were all collected from the same locations for each respective soil type, to ensure equal presentation context and material across experiments. Raw material (ca. 200 g) of each soil type was stored into closed sterile plastic bags, kept at room temperature. The four samples (ca. 2 g each) were placed at the corners of a plastic tray and presented to a single individual (for a total of 108 unique individuals) for at least one minute (unless the individual avoided or rejected the setup). Individuals were selected opportunistically, depending on their availability, given that they cannot be confined to a small space or separated from others. This resulted in 16 individuals being tested twice (Table S5), all being adults, among which only 3 interacted briefly with the set-up at least once, and none tried to taste or ingest any soil type. Among these 16 individuals tested twice, only 6 were tested within the same field season, while 10 were tested in different seasons (2023 and 2025).

Soil positions and sides of tray presented to the individuals were randomized for each trial. We made an effort to consistently approach and present the set-up to each individual with similar angles related to the position of the individual (in front of them, rather than on their side). The plastic tray with the samples was deposited in front of the individual at arm reach distance, on the substrate used by the macaque (floor or walls). Experimenters always deposited the tray with their right hand and stepped back few meters immediately to video record the trial. We filmed trials with a Canon EOS110D or GoPro 360 Max. For each experiment, we recorded the presentation order of soil types (which soil type was the closest to the individuals) and duration, whether the individual interacted with any soil, which type was first contacted, and whether it was tasted or ingested.

### Researcher’s survey

Given the limited published reports of geophagy in Barbary macaques, including Gibraltar, we aimed to assess whether this behavior is unique to this population. Using the published literature, we identified researchers and study sites working on Barbary macaques and contacted corresponding authors to ask whether they had ever observed geophagy at their sites, which age-classes were involved, whether the environment was anthropized, and how frequently it occurred. We contacted 63 authors across 29 study sites, including captive (*N* = 3), semi-captive (*N* = 5) and free-ranging (*N* = 21, including Gibraltar) populations. In total, 44 authors responded (70%), representing 26 sites (3 captive, 4 semi-captive and 19 free-ranging). Study duration varies widely across sites, which must be considered when interpreting observation rates, and caution is needed when inferring geophagy prevalence because soil availability was not systematically surveyed. Rates of geophagy reported to us shall also be taken with caution due to the lack of quantification in the vast majority of these reports. We therefore extrapolate the information given to us into the broad categories rare (occurring monthly or less), occasional (occurring weekly), frequent (occurring more than 2 times a week), and very frequent (occurring at least once a day) used by^3^. Full details on contacted researchers, study sites, and study periods are provided in the Supplementary Information (Data S1 – S2). We provide a descriptive analysis of these reports in the Results section.

### Statistical analysis

We used the focal follows to test two aspects of tourist-derived food consumption. First, a binomial GLMM assessed the likelihood of ingesting tourist-derived food (0 = none, 1 = ingestion during the focal follow). Second, a zero-inflated negative binomial GLMM modelled the number of tourist-derived food ingestion events per follow. The latter included total observation time as an offset term and assumed a constant zero-inflation probability. For both models, fixed effects were anthropogenic pressure, non-transformed food feeding time, sex (female, male) and season (winter, summer), with group and dates as random effects. We did not consider the age of individuals as focal follows were done only on adults, and adult age estimations were not consistently feasible. We were not able to compute dominance hierarchy data to include rank as a control variable, given that the position in the hierarchy may influence access to food resources. This is because accurate information on the identity of individuals interacting agonistically, from which rank data can be computed, have not been collected consistently and accurately during the study period used in this study. We also ran separate binomial and negative binomial GLMs to test for group differences in tourist-derived food consumption.

To assess the determinants of geophagy, we first fitted a binomial GLMM to the focal follow dataset. Fixed effects were anthropogenic pressure, non-transformed food feeding time, tourist-derived food feeding time, terra rossa outcrop density, sex, and season; group and dates were random effects. The response variable was whether geophagy occurred (0/1) during the follow.

Because most geophagy events occurred outside focal follows, we conducted a second analysis at the seasonal scale on the seasonal dataset (*N* = 47), which was analyzed with a binomial GLMM for the likelihood of geophagy and a zero-inflated negative binomial GLMM for event counts, with observation time as an offset term and constant zero inflation.

Regarding the soil presentation experiments, we analyzed the effect of group on the likelihood of first interacting with each soil type and on the likelihood of ingesting/tasting each type using binomial logistic regressions controlling for presentation order. Due to large differences in number of trials (Table S5) and interaction rates (AD 37.5%; RAW 38.4%; CC 48.1%; OH 25%; RG 14.3%; JG 0%), statistical analyses were limited to AD, RAW and CC (at least 20 trials and 30% interaction rates). Because terra rossa and tar dominated individual choices, analyses focused on the likelihood of first interacting with, and ingesting/tasting these two types. Yellow soil and black earth had too few cases (9 of 39 first choices; 6 of 21 ingestions) for meaningful modeling. This approach reduced our analyzed dataset to a sample size of 38 (for 38 unique individuals – AD 15, CC 13, RAW 10), which is why, to avoid complexification of the models, we did not include age and sex-classes as predictors.

All continuous predictors were z-transformed (mean = 0, SD = 1) in all models. Variance inflation factors (“vif” in ‘*car*’) from linear models without random effects showed no collinearity. We fitted models in R using ‘lme4’ (v.1.1.28)^91^ and ‘glmmTMB’ (v.1.1.11)^92^. Initial models with random slopes of the fixed effects failed to converge, so we only used random intercepts. Model fit was assessed by likelihood ratio tests comparing full and null models. Checks for overdispersion, leverage, and stability showed no issues. Confidence intervals were obtained via parametric bootstrapping. Marginal and conditional R² values were calculated with ‘*MuMin*’ (v.1.48.11)^93^ and ‘*partR2*’ (v.2.9.2)^94^. *Post-hoc* group comparisons used Tukey-adjusted least-squares means in “emmeans” (v.1.11.2). All tests were two-tailed, with significance at *p* < 0.05 and trends at 0.05 ≤ *p* < 0.10.

## Supplementary Information

Below is the link to the electronic supplementary material.

Supplementary Material 1

Supplementary Material 2

Supplementary Material 3

Supplementary Material 4

Supplementary Material 5

Supplementary Material 6

Supplementary Material 7

Supplementary Material 8

## Data Availability

All datasets used in the analyses are available on Dryad (doi:10.5061/dryad.j6q573nv0).
