# Supplementary material for: Geophagy in Gibraltar Barbary macaques is a primate tradition anthropogenically induced
Source: Sci Rep. 2026 Mar 19;16:13139. doi: 10.1038/s41598-026-44607-0 (PMC13102948; doi:10.1038/s41598-026-44607-0)
Supplement: Supplementary file 8 — Supplementary Material 8 [file 41598_2026_44607_MOESM8_ESM.pdf]

Supplementary Information:

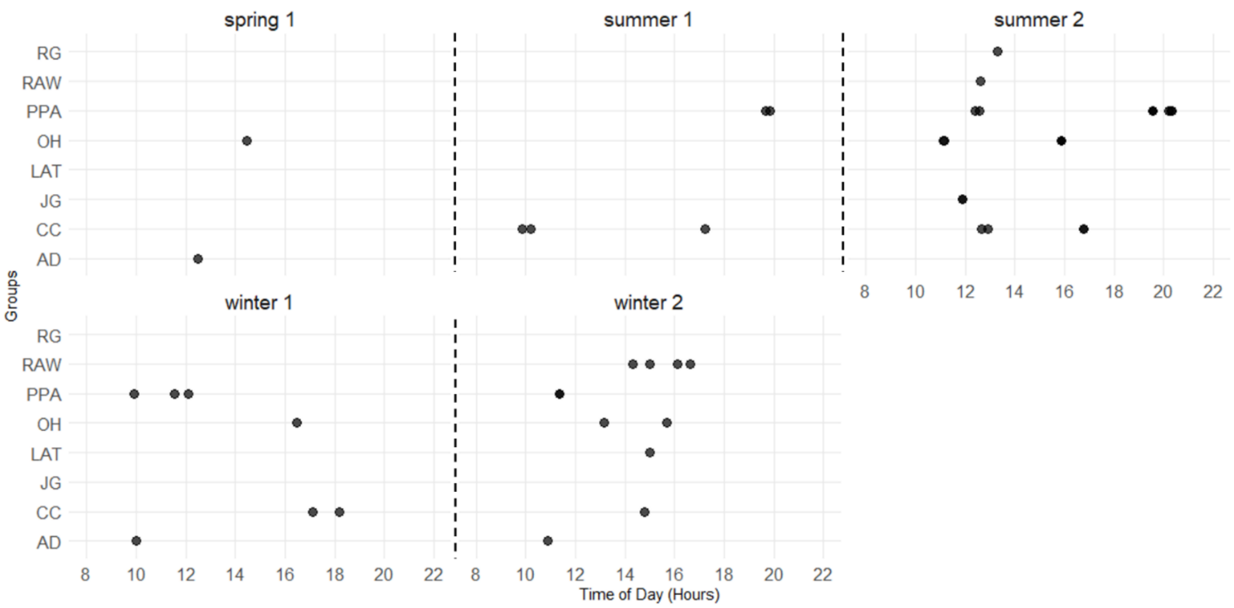

Fig. S1. Distribution of geophagy events across the various field seasons, for each group, as function of the time of the day. AD: Apes Den; CC: Cable Car; JG: Jew's Gate; LAT: Lathbury Barracks; OH: O'Hara; PPA: Prince Phillip's Arch; RAW: Royal Anglian Way; RG: Rock Gun.

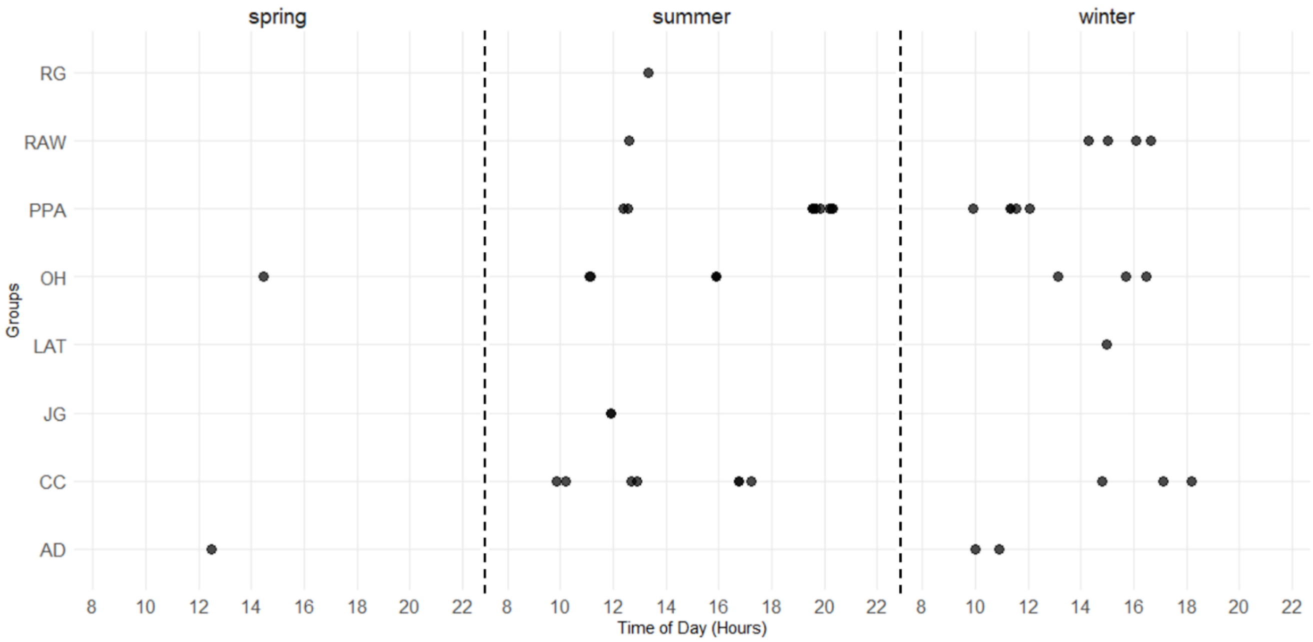

Fig. S2. **Distribution of geophagy events across the pooled field seasons, for each group, as function of the time of the day.** AD: Apes Den; CC: Cable Car; JG: Jew's Gate; LAT: Lathbury Barracks; OH: O'Hara; PPA: Prince Phillip's Arch; RAW: Royal Anglian Way; RG: Rock Gun.

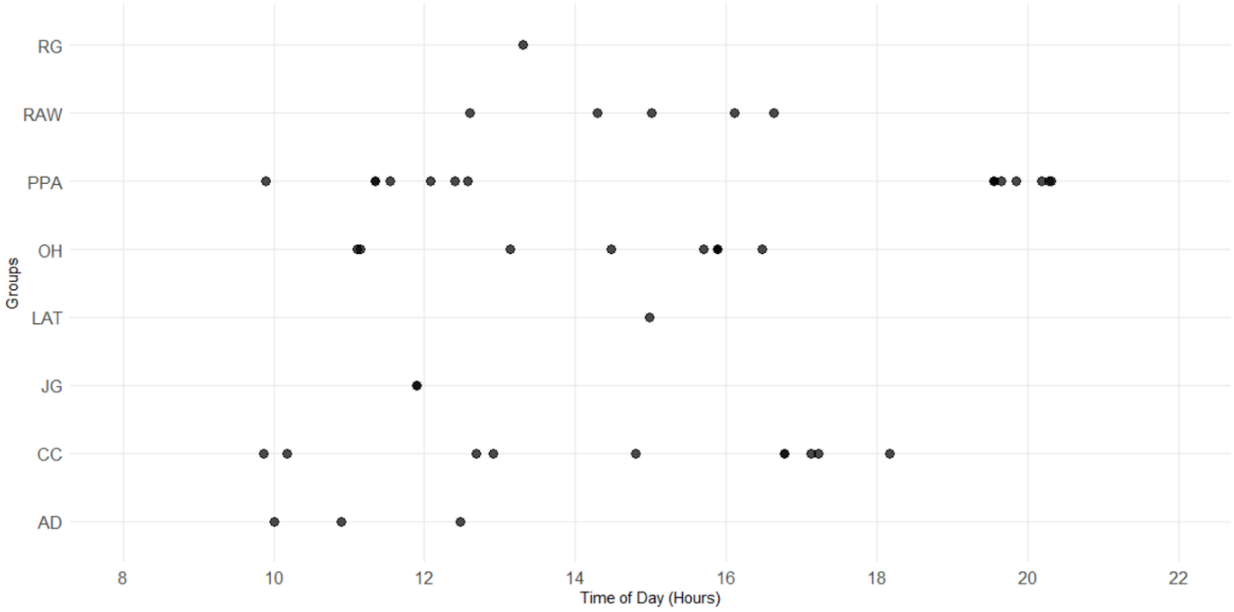

Fig. S3. Distribution of geophagy events for each group, as function of the time of the day. AD: Apes Den; CC: Cable Car; JG: Jew's Gate; LAT: Lathbury Barracks; OH: O'Hara; PPA: Prince Phillip's Arch; RAW: Royal Anglian Way; RG: Rock Gun.

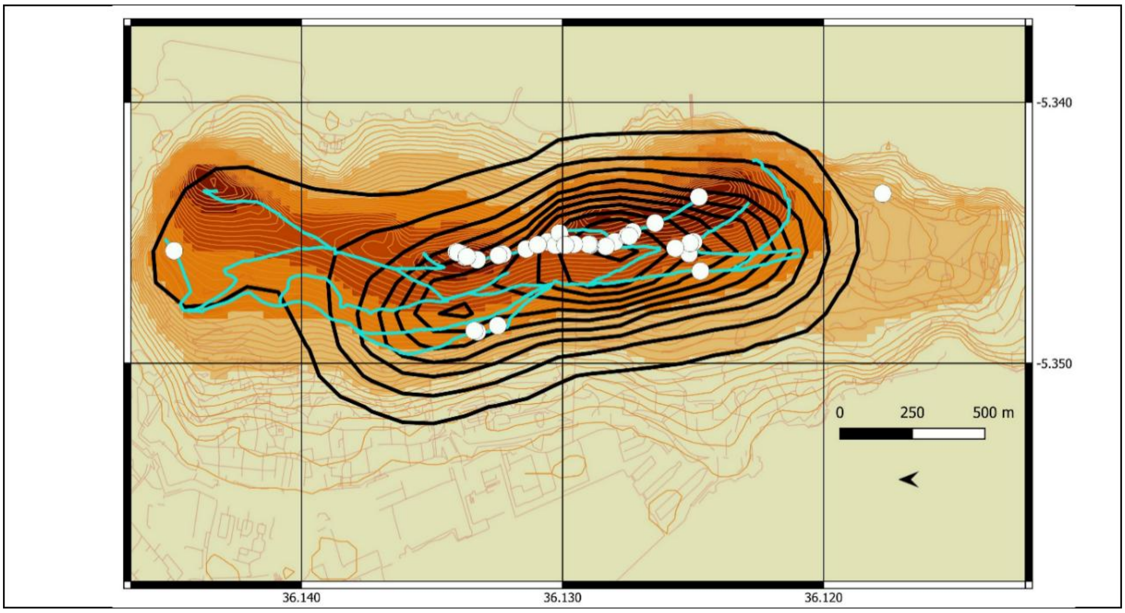

Fig. S4. Kernel distributions of terra rossa outcrops across the landscape, depicted with black lines, with higher outcrop density in the central areas. The elevation gradient appears in shades of orange (darker colors are higher); light blue lines correspond to the trails and paths used to survey outcrops; white dots correspond to the recorded events of geophagy.

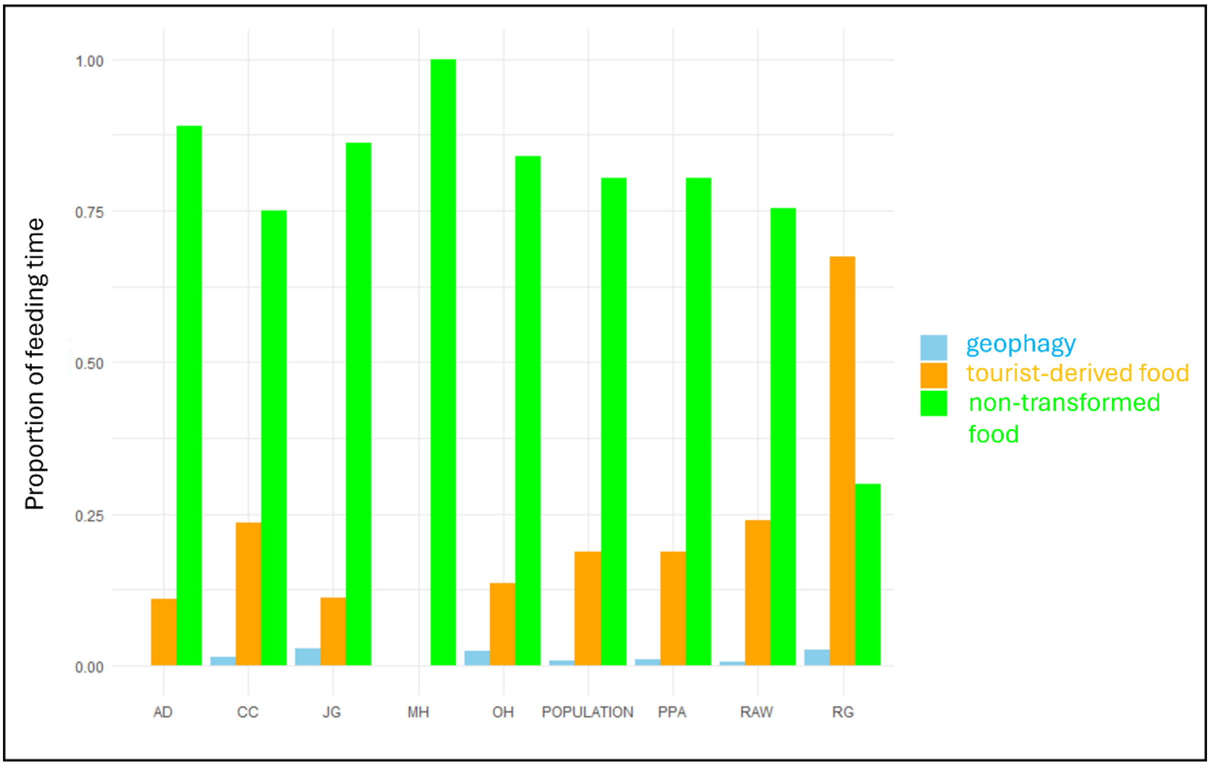

Fig. S5. Distributions, across the groups, of the proportion of feeding time (extracted from focal follows) spent on geophagy, tourist-derived food, and non-transformed food. AD: Apes Den; CC: Cable Car; JG: Jew's Gate; MH: Middle Hill; OH: O'Hara; PPA: Prince Phillip's Arch; RAW: Royal Anglian Way; RG: Rock Gun. Note the absence of geophagy for AD, since the 3 events in this group were observed outside of focal follows .

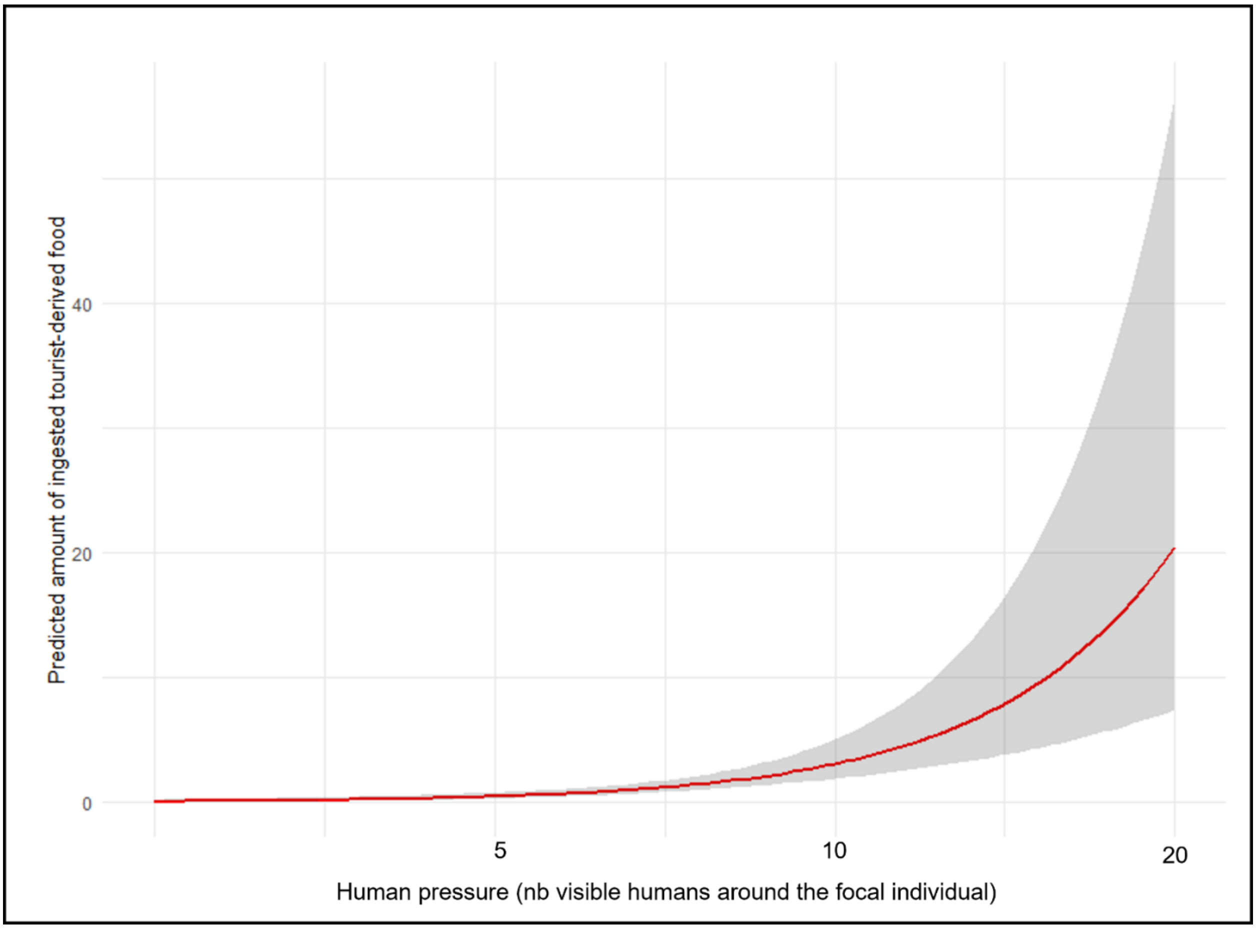

Fig. S6. Effect of anthropogenic pressure on the amount of ingested tourist-derived food. Model line is depicted in red, with the shaded area represents the 95% confidence intervals.

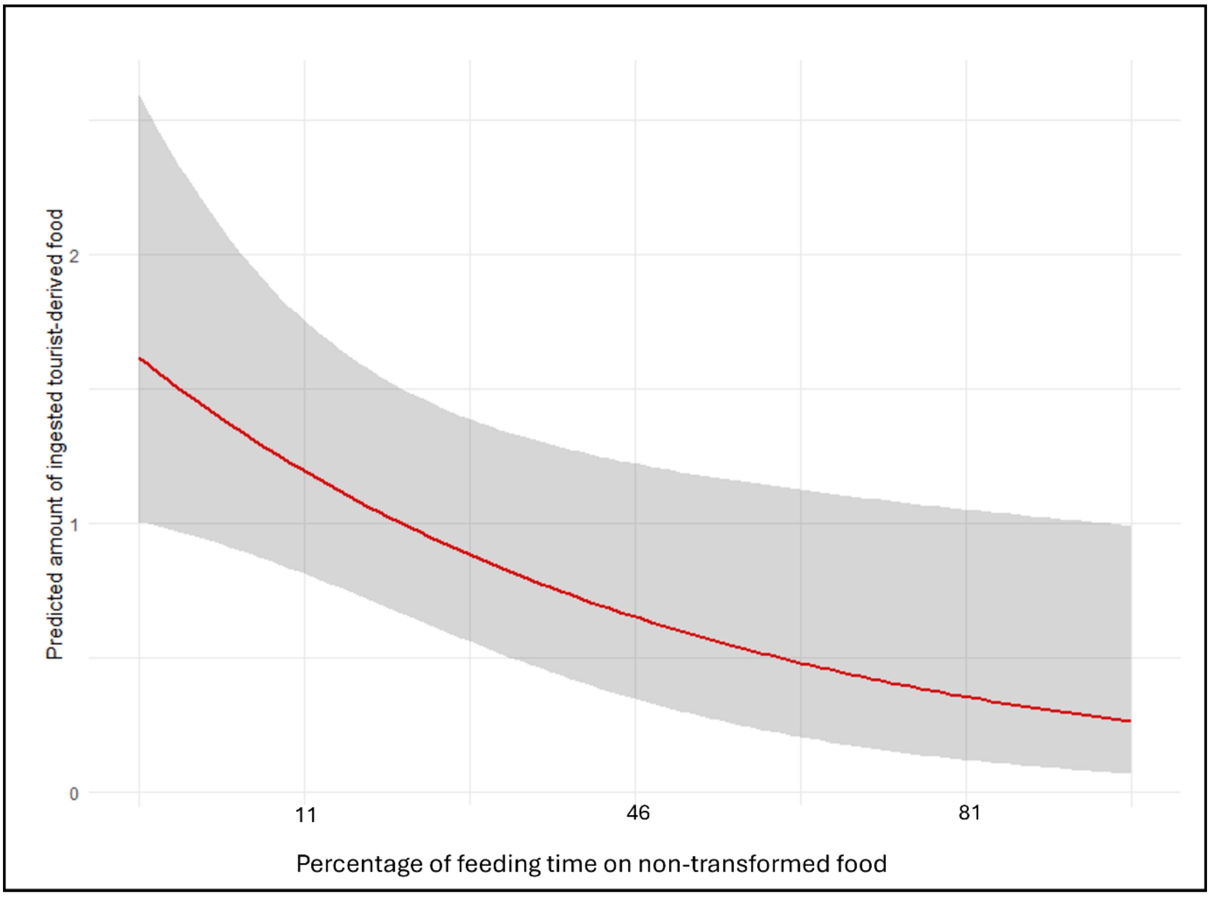

Fig. S7. Effect of the percentage of feeding time on non-transformed food, on the amount of ingested tourist-derived food. Model line is depicted in red, with the shaded area represents the 95% confidence intervals.

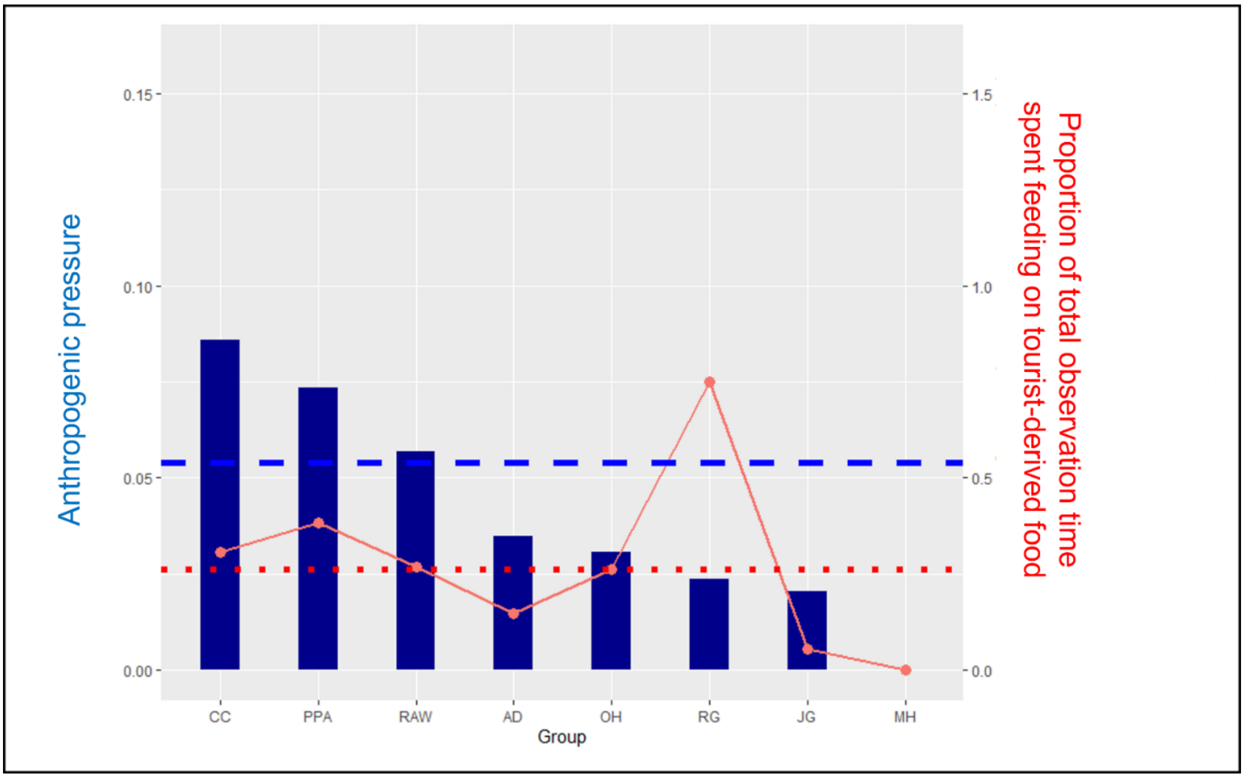

Fig. S8. Relationship per observed group, across seasons, between anthropogenic pressure (dark blue bars) and rates of ingestion of tourist-derived food (red dots). The horizontal blue dashed line represents the average anthropogenic pressure value for the whole population; the horizontal red dashed line represents the average proportion of observation time spent feeding on tourist-derived food for the whole population. Note the high rate of tourist-derived food feeding proportion for Rock Gun which is driven by one outlier focal follow including 19 bread-feeding records out of 55 observations (34.5% of total observation time), immediately followed by the only case of geophagy observed in that group.

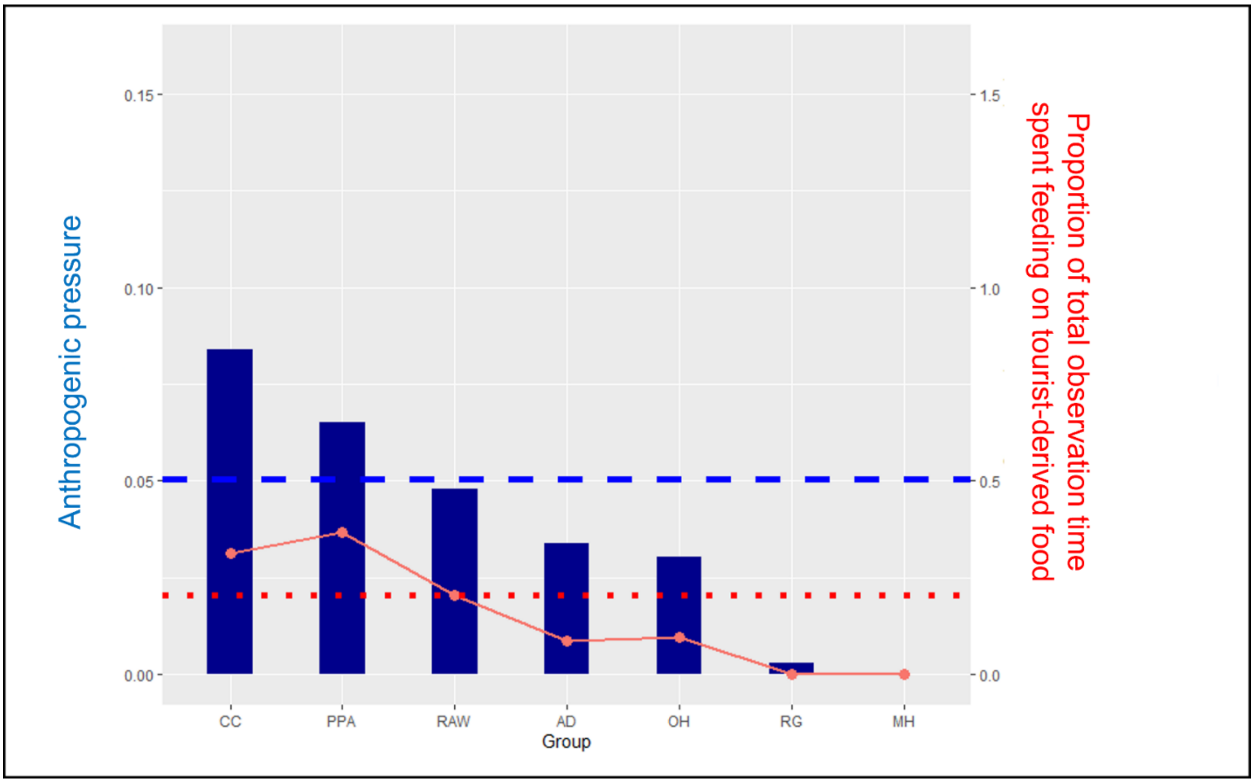

Fig. S9. Relationship per observed group, in winter, between anthropogenic pressure (dark blue bars) and rates of ingestion of tourist-derived food (red dots). The horizontal blue dashed line represents the average anthropogenic pressure value for the whole population; the horizontal red dashed line represents the average proportion of observation time spent feeding on tourist-derived food for the whole population.

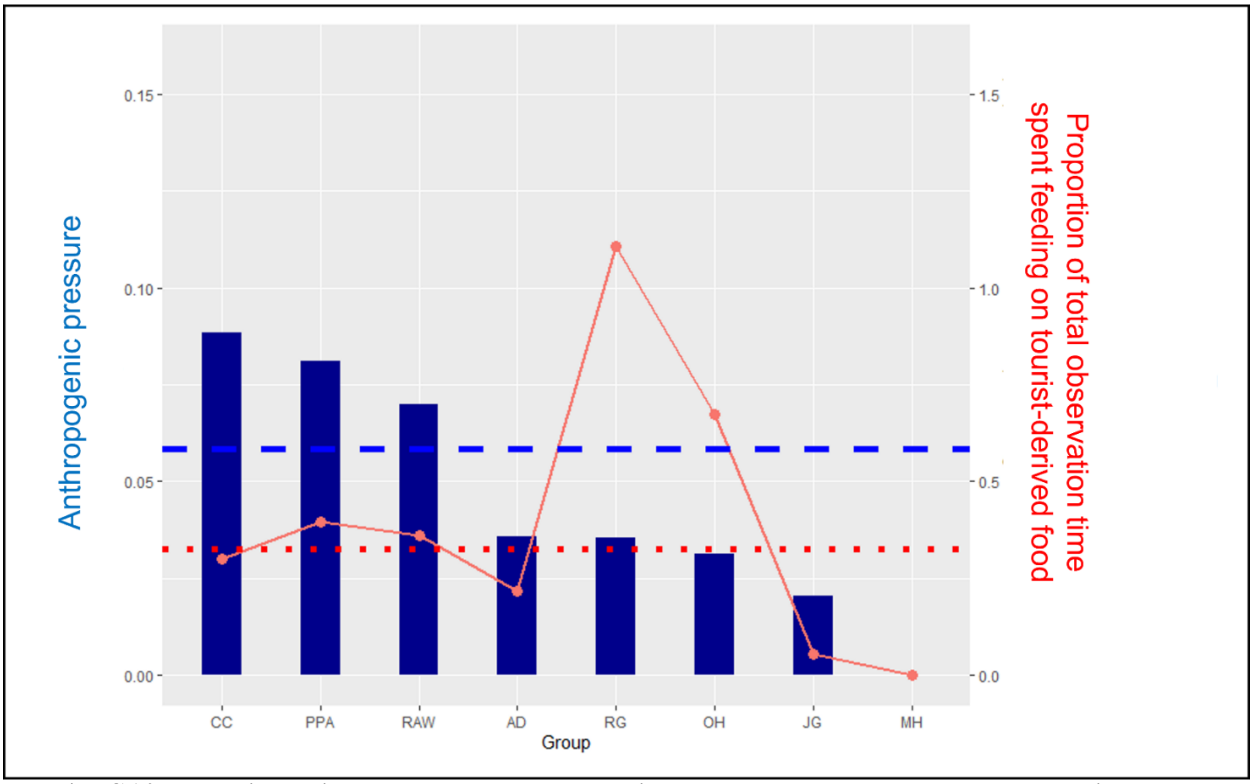

Fig. S10. Relationship per observed group, in summer, between anthropogenic pressure (dark blue bars) and rates of ingestion of tourist-derived food (red dots). The horizontal blue dashed line represents the average anthropogenic pressure value for the whole population; the horizontal red dashed line represents the average proportion of observation time spent feeding on tourist-derived food for the whole population.

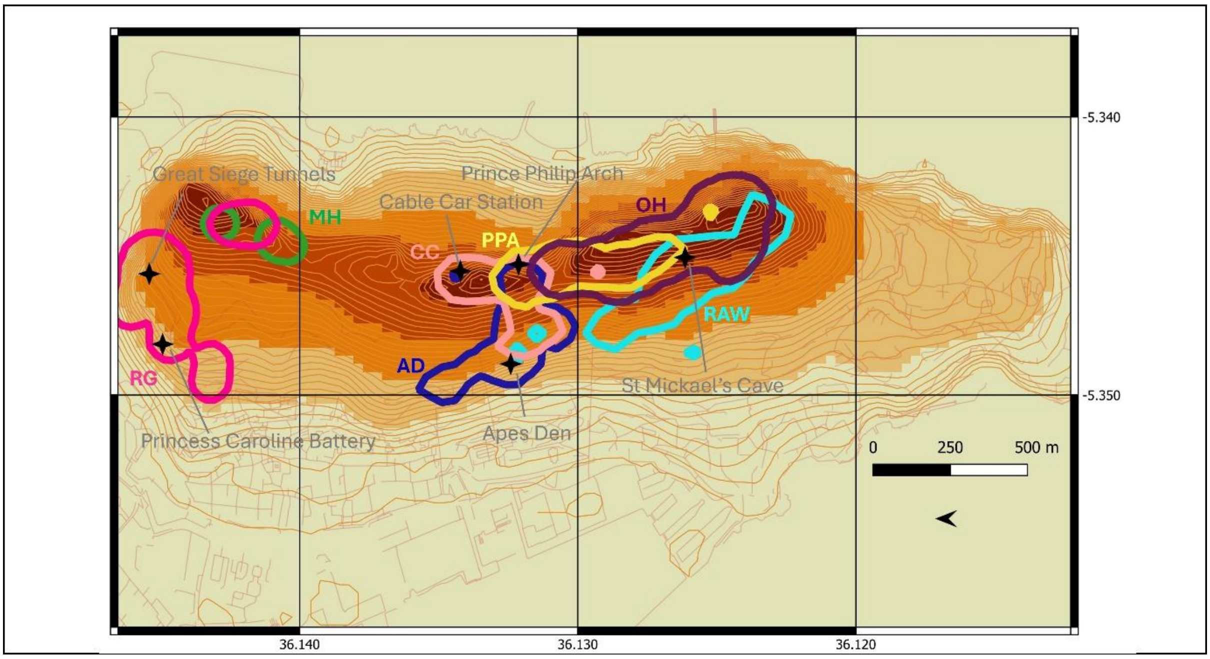

Fig. S11. Main touristic sites (black stars). The elevation gradient appears in shades of orange (darker colors are higher), thick lines delineate the home-ranges of the main study groups, delimited by 95% kernel utilization distributions: Apes Den (AD) in dark blue; Royal Anglian Way (RAW) in light blue; Cable Car (CC) in light pink; Prince Philip Arch (PPA) in yellow; O'Hara (OH) in purple; Middle Hill (MH) in green; Rock Gun (RG) in dark pink. This map has been created using QGIS Desktop 3.38.3 Grenoble https://www.qgis.org

Group	Total nb events	Terra rossa	Yellow soil	Black soil	Tar
**AD**	3	0	0	1	2
**CC**	10	10	0	0	0
**RAW**	5	5	0	0	0
**OH**	9	9	0	0	0
**PPA**	15	11	3	1	0
**RG**	1	1	0	0	0
**JG**	2	2	0	0	0
**MH**	0	0	0	0	0
**LAT**	1	0	0	1	0
**Population**	46	38	3	3	2

Table S1. Distribution of soil types observed during geophagy events across the study groups.

**Group**	**Total nb events**	**Adult male**	**Adult female**	**Subadult male**	**Subadult female**	**Juvenile male**	**Juvenile female**	**Infant male**	**Infant female**
**AD**	3	1	1	0	0	1	0	0	0
**CC**	10	3	7	0	0	0	0	0	0
**RAW**	5	1	4	0	0	0	0	0	0
**OH**	9	1	6	0	1	0	0	1	0
**PPA**	15	5	7	0	3	0	0	0	0
**RG**	1	1	0	0	0	0	0	0	0
**JG**	2	0	0	0	0	0	0	0	0
**MH**	0	0	0	0	0	0	0	0	0
**LAT**	1	0	0	0	0	1	0	0	0
**Population**	46	12	27	0	4	2	0	1	0
**Percentage**	100	26.08	58.70	0	8.70	4.35	0	2.17	0

Table S2. Summary of the demography of geophagy events across the study groups.

Terms	Estimate	SE	Z	95% CI	P
Intercept	-0.616	0.230	-2.669	-1.101; -0.162	NA
Anthropogenic pressure*, †	0.914	0.144	6.318	0.643; 1.212	**< 0.001**
Non-transformed food feeding time*, †	-0.233	0.144	-1.610	-0.532; 0.038	0.107
Sex – male*, ‡	-0.296	0.262	-1.129	-0.811; 0.219	0.258
Season – winter*, §	-0.514	0.259	-1.985	-1.026; -0.006	**0.047**

Determinants of tourist-derived food ingestion. Marginal effect sizes (R^2), counting for the variance explained by fixed effects, was 0.245, while conditional R2, counting for the variance of both fixed and random effects, was 0.252; * test predictor; † z-transformed; ‡ reference level is female; § reference level is summer; p-values in bold indicate a statistically significant effect ($p < 0.05$). Maximum VIF: 1.032.

Table S3. Determinants of the likelihood to ingest tourist-derived food. Results from a binomial GLMM.

Terms	Conditional model (log-odds of success)	SE	Z	95% CI	P	Zero-inflation model (log-odds of excess of zeros)	SE	Z
Intercept	-3.925	0.237	-16.520	-4.391; -3.460	NA	-12.88	319.39	-0.04
Anthropogenic pressure*, †	0.946	0.161	5.868	0.630; 1.262	**< 0.001**			
Non-transformed food feeding time*, †	-0.302	0.131	-2.302	-0.560; -0.044	**0.021**			
Sex – male*, ‡	0.273	0.253	1.079	-0.223; 0.771	0.280			
Season – winter*, §	-0.563	0.246	-2.288	-1.045; -0.080	**0.022**			

Determinants of tourist-derived food ingestion. Marginal effect sizes (R^2), counting for the variance explained by fixed effects, was 0.208, while conditional R2, counting for the variance of both fixed and random effects, was 0.208; * test predictor; † z-transformed; ‡ reference level is female; § reference level is summer; p-values in bold indicate a statistically significant effect ($p < 0.05$). Maximum VIF: 1.044.

Table S4. Determinants of the amount of ingested tourist-derived food. Results from a zero-inflated negative binomial GLMM.

Group	Nb presentations (nb with interaction)	Nb Adult/subadult males tested (nb interacted) – [nb unique tested]	Nb Adult/subadult females tested (nb interacted) – [nb unique tested]	Nb Juveniles tested (nb interacted) – [nb unique tested]	Nb Infants tested (nb interacted) – [nb unique tested]
AD	40 (15)	7 (0) – [7]	16 (1) – [12]	15 (12) – [15]	2 (2) – [2]
CC	27 (13)	5 (1) – [5]	12 (3) – [8]	7 (6) – [7]	3 (3) – [3]
RAW	26 (10)	10 (0) – [6]	9 (4) – [9]	7 (6) – [7]	0 (0)
OH	4 (1)	0 (0)	4 (1) – [4]	0 (0)	0 (0)
PPA	19 (2)	5 (0) – [4]	11 (1) – [8]	2 (1) – [2]	1 (0) – [1]
RG	7 (1)	2 (0) – [2]	1 (0) – [1]	4 (1) – [4]	0 (0)
JG	1 (0)	0 (0)	1 (0) – [1]	0 (0)	0 (0)
all	124 (42)	29 (1) – [24]	54 (10) – [43]	35 – [35]	6 (5) – [6]

Table S5. Summary of the soil presentation experiments including the number of tested individuals across the groups. AD: Apes Den; CC: Cable Car; RAW: Royal Anglian Way; OH: O'Hara; PPA: Prince Philip Arch; RG: Rock Gun; JG: Jew's Gate.

**Terms**	**Estimate**	**SE**	**Z**	**95% CI**	**P**
Intercept	-0.506	0.971	-0.522	-2.520; 1.396	NA
Group CC*,†	-2.356	1.164	-2.024	-5.387; -0.386	**0.043**
Group RAW*,†	-1.957	1.185	-1.651	-5.011; 0.069	*0.098*
Order presentation tar‡	0.254	0.561	0.454	-0.869; 1.490	0.650

Marginal effect sizes (R^2), counting for the variance explained by fixed effects, was 0.275. * test predictor; † reference level is Apes Den AD; ‡ control predictor; p-values in bold indicate a statistically significant effect ($p < 0.05$), p-values in *italic* indicate a trend ($p < 0.10$).

Table S6. Determinants of the *likelihood to ingest tar* during the soil presentation experiments. Results are based on a dataset comprising all experiments done on Apes Den (AD), Royal Anglian Way (RAW) and Cable Car (CC), where individuals interacted with the protocol (N=38). Results are generated from a generalized linear model with binomial structure.

**Group**	Est. number of individuals	Est. number of adult & subadult males	Est. number of adult & subadult females
**Apes Den AD**	ca. 40	8-10	17
**Cable Car CC**	ca. 45	10-13	22
**Royal Anglian Way RAW**	27	8	14
**O'Hara OH**	12	3-4	7
**Prince Philip Arch PPA**	17	4-5	11
**Rock Gun RG**	25	7	9
**Jew's Gate JG**	3-4	0	3-4
**Middle Hill MH**	24	6	14
**Lathbury Barracks LAT**	ca. 30	8	8
**Population**	ca. 220	54-61	105-106

Table S7. Demography of the study groups.

Movie S1 (separate file).

Adult female from Royal Anglian Way group, feeding on a terra rossa outcrop along the roadside. Video taken on the 7 January 2024. Credit: Martin Nicourt/Gibraltar Macaques Project.

Movie S2 (separate file).

Adult female from Cable Car group, feeding on a terra rossa outcrop at Cable Car station. Video taken on the 12 January 2024. Credit: Martin Nicourt/Gibraltar Macaques Project.

Movie S3 (separate file).

Adult male from Prince Philip Arch group, feeding on a terra rossa outcrop along the roadside. Video taken on the 8 December 2023. Credit: Sylvain Lemoine/Gibraltar Macaques Project.

Movie S4 (separate file).

Adult female from Prince Philip Arch group, feeding on the same terra rossa outcrop along the roadside than the adult male from Movie S3, immediately afterwards. Video taken on the 8 December 2023. Credit: Sylvain Lemoine/Gibraltar Macaques Project.

Movie S5 (separate file).

Adult female from Cable Car group, feeding on a terra rossa outcrop at Cable Car station, in presence of one one-year and one two-years old juveniles observing the activity. Video taken on the 14 August 2024. Credit: Sylvain Lemoine/Gibraltar Macaques Project.

Data S1. (separate file)

Summary of researchers contacted, study sites, periods of study, site types and whether researchers responded to the survey.

Data S2 (separate file)

Summary of study sites reported in Figure 5, geographic coordinates, whether geophagy was observed at these sites, sources of responding authors, study periods of responding authors, and provided geophagy details.
